# Regulation of Gonad Morphogenesis in *Drosophila melanogaster* by BTB Family Transcription Factors

**DOI:** 10.1371/journal.pone.0167283

**Published:** 2016-11-29

**Authors:** Diane Silva, Kenneth W. Olsen, Magdalena N. Bednarz, Andrew Droste, Christopher P. Lenkeit, Edwin Chaharbakhshi, Emily R. Temple-Wood, Jennifer C. Jemc

**Affiliations:** 1 Department of Biology, Loyola University Chicago, Chicago, IL, United States of America; 2 Department of Chemistry and Biochemistry, Loyola University Chicago, Chicago, IL, United States of America; National Cancer Institute, UNITED STATES

## Abstract

During embryogenesis, primordial germ cells (PGCs) and somatic gonadal precursor cells (SGPs) migrate and coalesce to form the early gonad. A failure of the PGCs and SGPs to form a gonad with the proper architecture not only affects germ cell development, but can also lead to infertility. Therefore, it is critical to identify the molecular mechanisms that function within both the PGCs and SGPs to promote gonad morphogenesis. We have characterized the phenotypes of two genes, *longitudinals lacking (lola)* and *ribbon (rib)*, that are required for the coalescence and compaction of the embryonic gonad in *Drosophila melanogaster*. *rib* and *lola* are expressed in the SGPs of the developing gonad, and genetic interaction analysis suggests these proteins cooperate to regulate gonad development. Both genes encode proteins with DNA binding motifs and a conserved protein-protein interaction domain, known as the Broad complex, Tramtrack, Bric-à-brac (BTB) domain. Through molecular modeling and yeast-two hybrid studies, we demonstrate that Rib and Lola homo- and heterodimerize via their BTB domains. In addition, analysis of the colocalization of Rib and Lola with marks of transcriptional activation and repression on polytene chromosomes reveals that Rib and Lola colocalize with both repressive and activating marks and with each other. While previous studies have identified Rib and Lola targets in other tissues, we find that Rib and Lola are likely to function via different downstream targets in the gonad. These results suggest that Rib and Lola act as dual-function transcription factors to cooperatively regulate embryonic gonad morphogenesis.

## Introduction

**Fig 1:**
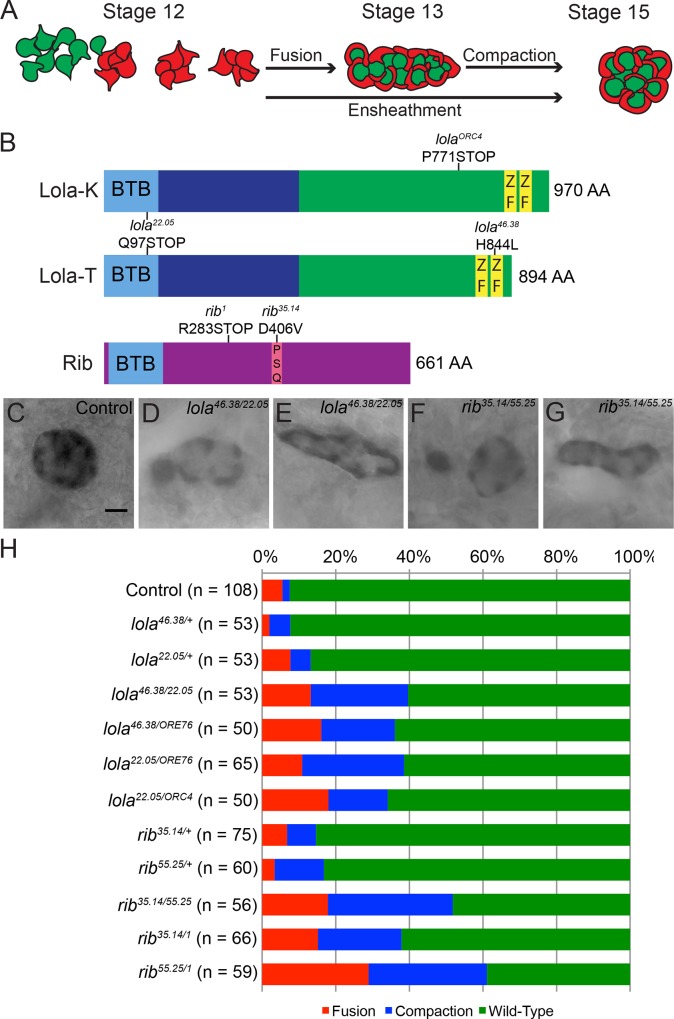
*lola* and *rib* mutants exhibit defects in gonad morphogenesis. (A) Schematic of the stages of embryonic gonad formation. At stage 12 SGP (red) clusters and PGCs (green) begin to intermingle. At stage 13 SGP clusters fuse and coalesce with the PGCs, sending out extensions around PGCs to ensheath them. By the end of stage 15 SGPs and PGCs compact to form a spherical gonad. (B) Molecular structure of Lola and Rib with BTB domains (light blue), Lola common region (dark blue), zinc finger DNA binding motifs (ZF; yellow), pipsqueak DNA binding motif (PSQ; pink), and the corresponding mutations found in alleles used in this study. (C-G) Stage 15 mutant embryos immunostained for the *68-77*-*lacZ* enhancer trap, which labels the cytoplasm of SGPs for analysis of gonad morphology. Posterior to the right. (C) Control embryos expressing the *68-77-lacZ* enhancer trap. Scale bar: 10μm. (D-E) *lola*^*46*.*38/22*.*05*^ embryonic gonads exhibiting fusion (D) and compaction (E) defects. (E-F) *rib*^*35*.*14/55*.*25*^ embryonic gonads exhibiting (F) fusion and (G) compaction defects. (H) Quantification of the frequency of gonad phenotypes observed in control, *lola* mutant and *rib* mutant embryos. Gonad morphology was assessed using the *68-77-*lacZ enhancer trap. The following phenotypes were scored: fusion (red), compaction (blue) and wild-type (green). A chi-square test was performed to test the null hypothesis that the phenotypic ratios will be the same across all genotypes. Results allow us to reject the null hypothesis: *Χ*^2^_*28*, *0*.*05*_ = 118.95, p<0.001.

Organ development depends upon the specification, migration, and interaction of multiple cell types, which give structure and function to that organ. Failure to execute any of these steps during development can result in birth defects or even lethality. The embryonic gonad provides an excellent model to study the genes that regulate cell migration and cell-cell interactions during organogenesis, as it is formed from two primary cell types: the primordial germ cells (PGCs) and the somatic gonadal precursors (SGPs) [1, 2]. PGCs are formed at the posterior end of the embryo at stage 4–5 and remain there until stage 7, when the midgut invaginates and passively pulls the PGCs with it [2, 3]. The PGCs begin active migration through the midgut epithelium toward the mesoderm during germ band elongation at stage 9 [3, 4]. The SGPs are specified bilaterally in three clusters from the mesodermal layer of abdominal parasegments 10–12 at stage 11 [1, 2]. During stage 12, the PGCs migrate bilaterally and begin to intermingle with the SGPs as the germ band retracts [5]. By the end of germ band retraction at stage 13 the three SGP clusters and the PGCs coalesce into an elongated gonad on each side of the developing embryo (Fig 1A) [5]. During stage 13 SGPs also begin to ensheath the PGCs by sending out membrane extensions, which persist throughout gonad development and are critical for proper germ cell development [6–9]. Following gonad coalescence, SGPs and PGCs compact to form a spherical gonad by stage 15 of embryogenesis (Fig 1A) [5]. Previous studies have identified many genes that are critical for PGC migration, gonad coalescence and compaction, and ensheathment [10, 11]; however, understanding of this complex process is far from complete. In this paper, we describe the role of two genes, *ribbon (rib)* and *longitudinals lacking (lola)*, in embryonic gonad morphogenesis.

Molecularly, Rib and Lola belong to the BTB/POZ (Broad Complex, Tramtrack and Bric à Brac/Pox Virus and Zinc finger) family of proteins. These proteins include a conserved BTB domain, which has been demonstrated to mediate protein-protein interactions [12–16]. The BTB domain is located at the amino (N)-terminus where it mediates homo- and heterodimerization, as well as multimerization with other BTB and non-BTB domain-containing proteins [14, 17, 18]. Many BTB domain-containing proteins, including Rib and Lola, contain an N-terminal extension of the BTB domain, which plays an important role in stabilizing BTB domain interactions [18]. Frequently, the BTB domain is observed in combination with DNA-binding motifs, and studies have demonstrated that many of these BTB family proteins function as transcriptional regulators [19–22]. In some cases, the BTB domain has been shown to interact with transcriptional repressors and activators, further supporting this regulatory role [17, 23–26].

Both Lola and Rib contain DNA binding motifs in addition to a BTB domain. The *lola* gene locus encodes at least 20 protein isoforms generated by alternative splicing [27, 28]. While all protein isoforms contain a common N-terminal region, which includes the BTB domain, their carboxy (C)-terminal domain structure varies [27–29]. Of the 20 identified protein isoforms, there are only 3 isoforms that lack a zinc finger motif at the C-terminus, while most isoforms contain two zinc fingers, as shown for isoforms T and K (Fig 1B) [28]. Variability in the sequence of these zinc fingers and analysis of the DNA binding specificity of different Lola isoforms suggests that different isoforms have different DNA binding specificities [28, 30]. In addition, yeast two-hybrid studies reveal that different Lola isoforms are capable of heterodimerization, thereby increasing the variability of potential Lola binding sites [31, 32]. The presence of zinc fingers implies that Lola may function as a transcriptional regulator, and previous studies have demonstrated the ability of Lola to repress expression of the *copia* retrotransposon in the embryonic central nervous system [19]. While Lola is hypothesized to function primarily as a transcriptional repressor, expression of *copia* appears reduced in the embryonic gonad of *lola* mutants [19]. These results suggest that Lola isoform function may be determined in part by its binding partners, or that different Lola isoforms regulate gene expression in different tissues. Gene expression profiling of *lola* null mutant embryos compared to controls has resulted in the identification of hundreds of genes that exhibit increased or decreased expression upon *lola* mutation, although the direct transcriptional regulation of these targets has not yet been demonstrated [33].

Unlike *lola*, which has at least 20 protein isoforms, the *rib* gene locus encodes three protein isoforms. All Rib isoforms have a DNA binding motif located C-terminal to the BTB domain that is referred to as the Pipsqueak (PSQ) motif (Fig 1B) [15, 16]. The PSQ motif is a 50-amino acid sequence that binds to DNA [34, 35]. Recent work has resulted in the identification of multiple Rib transcriptional targets in the salivary gland, some of which are repressed while others are activated [36]. These results suggest that Rib transcriptional activity may also depend on its interaction with different binding partners.

Both *rib* and *lola* were previously identified in genetic screens for mutants that affect early gonad formation [37, 38], and have been shown to function in other contexts to regulate organ morphogenesis. The *rib* gene was first identified in an ethyl methanesulfonate mutagenesis screen for larval cuticle abnormalities [39]. Subsequently, *rib* was shown to regulate cell migration and morphogenesis of the trachea and salivary glands [15, 16, 40]. When *rib* is mutated, the tracheal cell bodies and apical surface have severe migration defects and impaired morphogenesis, while the salivary glands fail to elongate [15, 16]. Recent work has demonstrated that Rib is required to promote the changes in cell shape and volume needed for salivary gland morphogenesis [36]. Molecular studies of the downstream effectors of Rib are consistent with these cellular functions. *rib* mutants exhibit reduced levels of Crumbs (Crb), an apical membrane protein that functions in epithelial cell polarity and in apical membrane growth [41, 42]. Rib also regulates the activity of Moesin (Moe), the only Ezrin-Radixin-Moesin (ERM) family protein in *Drosophila*, which plays a role in linking the plasma membrane to the actin cytoskeleton [42]. In *rib* mutants, levels of active, phosphorylated Moe are increased, suggesting that down-regulation of Moe activity is required for salivary gland and tracheal morphogenesis [42]. Finally, levels of the recycling endosomal protein Rab11 were reduced in *rib* mutants, consistent with an observed decrease in the number of apically-localized vesicles [42]. In the context of salivary gland and tracheal morphogenesis, Rib has also been demonstrated to genetically interact with another BTB protein, Lola like (Lolal) [42]. Given that Rib interacts with other BTB domain containing proteins such as Lolal, it is possible that Rib may interact with other BTB family proteins in a tissue-specific manner to regulate development of other tissues.

One candidate for this interaction is Lola. Previous studies have demonstrated a critical role for Lola in *Drosophila* nervous system development. *lola* is required for axon growth in the embryonic central nervous system (CNS), and mutation of *lola* results in axon pathfinding defects along the longitudinal tracts of the CNS [43]. In addition, Lola is required to prevent midline crossing of longitudinal axons [44]. In this context, Lola appears to up-regulate expression of the midline repellent protein, Slit, and its longitudinal axonal receptor Roundabout (Robo) [28, 44]. Previous studies have demonstrated that Lola is also required for embryonic gonad morphogenesis and for gametogenesis in the adult [22, 37, 38, 45].

Here, we have further characterized the role of Lola and Rib in embryonic gonad morphogenesis. Using genetic and physical interaction analysis, we have examined the relationship between Rib and Lola and our results suggest that they cooperate to promote gonad morphogenesis. Molecular modeling suggests that both Rib and Lola are capable of homo- and heterodimerization via their BTB domains. The colocalization of Rib and Lola with marks of transcriptional activation and repression and with each other at a subset of sites on polytene chromosomes suggests that Rib and Lola function as dual-function transcriptional regulators to regulate tissue morphogenesis.

## Materials and Methods

### Fly strains and genetics

The following stocks were acquired from the Bloomington *Drosophila* stock center (Indiana University, Bloomington, IN, USA): Oregon R, PBac{lola.GR-GFP.FLAG}VK00033, PBac{lola.I-GFP.FLAG}VK00033, *lola*^*ORE76*^ [43], and *rib*^*1*^ [16]. *lola*^*46*.*38*^, *lola*^*22*.*05*^, *rib*^*35*.*14*^, *rib*^*55*.*25*^ [37], *68-77*-*lacZ* [46], *forkhead*-Gal4 [47], and 24B-Gal4 [48] were acquired from Mark Van Doren. *lola*^*ORC4*^ [43] was a gift from Edward Giniger and *rib*^*1*^, UAS-*rib* [16] was a gift from Deborah Andrew. Balancer chromosomes carrying a transgene encoding Green Fluorescent Protein (*Kr*-Gal4, UAS-GFP) [49] or Yellow Fluorescent Protein (*dfd*-eYFP) [50] were used for genotyping.

### Immunohistochemistry and microscopy

Embryo fixation and immunostaining were performed as previously described [9] with the exception of immunostaining for Robo. For Robo immunostaining, embryos were fixed in 1.75 ml PCM (100mM Pipes pH 6.9, 1mM CaCl_2_, 2mM MgSO_4_), 0.25 ml 37% formaldehyde, and 8 ml heptane for 22 minutes. Embryos were washed in heptane, dried, and rehydrated in PBTx (1xPBS, 0.1% Triton X-100). Embryos were briefly sonicated to devitellinize them, washed, blocked, and immunostained in BBTx (1xPBS, 0.3% Triton-X-100, 1% BSA) with 5% normal goat serum. The following primary antibodies were used (dilution, source): chick-GFP (1:1000, Abcam); rabbit anti-GFP (1:2000, Torrey Pines Biolabs); mouse anti-GFP (1:50, Santa Cruz); rabbit anti-β-Galactosidase (1:1,000, Cappel); rat anti-Rib (1:50, Deborah Andrew [42]); rabbit anti-Lola (1:100, Edward Giniger [43]); guinea pig anti-Traffic Jam (1:1000, Mark Van Doren [9, 51]); rabbit anti-Vasa (1:200, Santa Cruz Biotechnology); rat anti-Vasa (1:50, Developmental Studies Hybridoma Bank (DSHB)); mouse anti-Eya (1:25, DSHB); rabbit anti-Vasa (1:200, Santa Cruz Biotechnology); mouse anti-Crb (1:10, DSHB); rabbit anti-phospho-Moe (1:200, Cell Signaling Technology); mouse anti-Robo (1:10, DSHB); mouse anti-SpirC3 (1:100, Susan Parkhurst [52]) and mouse anti-Fasciclin 3 (1:30, DSHB). Alexafluor 488, 546, and 633 conjugated secondary antibodies were used at 1:500 (Invitrogen) and mounted in DABCO (70% glycerol, 2.5% 1,4-diazabicyclo[2.2.2]octane, 10mM Tris-HCl pH 7.5) for immunofluorescence microscopy. For immunohistochemical staining, biotin conjugated secondaries (Jackson ImmunoResearch) were used at 1:5000, and the stain was developed using the ABC Elite kit (Vector Labs) using DAB (3′3′-diaminobenzidine) as a substrate (Vector Labs). These embryo stains were mounted in 80% glycerol/20% PBS. Embryos were staged according to their gut morphology. Fluorescently stained embryos were imaged on an Olympus Fluoview 1000 confocal microscope equipped with 488, 561 and 633 lasers using a PlanApo N 60x oil (NA 1.42) objective. All images are a single plane unless otherwise noted. Immunohistochemically stained embryos were imaged on a Zeiss AxioImager.M2 using an EC Plan-NEOFLUAR 40x oil (NA 1.3) objective. Images were processed using ImageJ software.

### Plasmid construction

For yeast two-hybrid analysis, DNA fragments were PCR amplified from LD16058 DNA (*rib*) and LD28033 DNA (*lola*) (Drosophila Genomics Resource Center), using the following primers: Rib-Ndel-Fwd 5’-CATGCATATGGGCGGCCCAACGGCG-3’, Rib-BamHI-Rev 5’-TGCAAGGATCCTATGATTGAACTTCATCAAGTTGTCGTACAGAC-3’, Lola-Ndel-Fwd 5’-CATGCATATGGATGACGATCAGCAGTTTTGTTTG-3’, and Lola-BamHI-Rev 5’-TGCA AGGATCCTTACTCCGCCGCCAGTGCG-3’. PCR fragments were cloned into the multiple cloning site of the pGADT7 and pGBKT7 vectors (Clontech) using NdeI and BamHI.

For the UAS-3xHA-*rib* transgene, DNA fragments were PCR amplified from LD16058 DNA (*rib*), using the following primers: Rib-FL-Reverse 5’-CAAGGGATCCGCGTTAATCA GTCGGCCCGGGCCTGAGCGT-3’, 3xHA-rib-Kozak 5’-CAAGGCGGCCGCGCCGCCACC ATGGGATACCCATACGATGTTCCAGATTACGCTTACCCATACGATGTTCCAGATTAC GCTTACCCATACGATGTTCCAGATTACGCTGGAGGAGGCGGCCCAACGGCGCCG -3’. PCR fragments were cloned into the pUASpB (a modified version of pUASP [53] with an attB site for phiC31-mediated integration) using NotI and BamHI. Transgenic flies were generated by integration of this construct into P{CARYP} attP40 [54, 55] by phiC31 integration by BestGene Inc. (Chino Hills, CA).

### Yeast-two hybrid interaction

The yeast strains Y2H-Gold and Y187 were transformed with pGADT7 and pGBKT7 vectors, respectively, by standard Lithium acetate-Tris-EDTA transformation and transformants were selected on SD-Leu or SD-Trp plates. The yeast two-hybrid was performed according to the Matchmaker^®^ Gold Yeast Two-Hybrid System User Manual (Clontech) with a few modifications. For yeast mating, individual yeast colonies were inoculated into 5 ml of YPDA in 250 ml Erlenmeyer flasks in the morning. In the mid/late afternoon, yeast growth was measured by a spectrophotometer to ensure similar culture density. The desired mating combinations were mixed with equal amounts of media in a 500 ml Erlenmeyer flask and then the volume was brought up to ~10 ml for incubation overnight at 30°C. After 20 hours, cultures were examined for the presence of zygotes in the media and plated on SD-Trp-Leu media to select for successful mating. Following successful mating, colonies were streaked on SD-Leu-Trp-His-Ade plates supplemented with X-α-Gal and Aureobasidin A for yeast two-hybrid analysis.

### Protein modeling

Evolutionarily related structures for the sequences of Lola and Rib were found by using Blast [56] and HHBlits [57] to search the SWISS-MODEL template library (SMTL version 2016-03-30, PDB release 2016-03-25) [58]. Homology models for the BTB domain dimers of Lola and Rib were constructed using SWISS-MODEL [59] using the crystal structure of the human Bach2 POZ domain (PDBid: 3OHV) [60] as a template. The initial dimer for Lola-Rib was modeled using one subunit from each of the homodimers. The three initial dimers were then refined using a combination of energy minimization and molecular dynamics. Each simulation box, containing one dimer, a TIP3 water box extending at least 10 Å beyond the protein in all directions and 0.15 M NaCl adjusted to neutralize the charge in the water box, was assembled using the molecular graphics program VMD [61]. The simulation box was then brought to equilibrium using the molecular dynamics program NAMD [62]. The equilibration procedure involved energy minimization with and without restraints on the protein coordinates (3000 steps each), slow heating from 10 to 310 K (30,000 steps), and then pressure and temperature equilibration using a Langevin piston (10,000 steps). Finally, unrestrained dynamics for 100,000 steps was done before data was acquired. The time step was 2 fs with every 150th step being saved in the trajectory for analysis. Periodic boundary conditions were used. The cutoffs for nonbonding (van der Waals and electrostatic) interactions were 12 Å. The switch distance was 10 Å, and 1.0 1–4 scaling factor was used. All calculations were done using CHARMM 27 parameters [63]. The van der Waals and electrostatic dimer interaction energy values presented in Table 1 were determined for the final 100 steps (30 ps) in each simulation and then averaged. All molecular graphics diagrams were generated using VMD [61].

**Table 1:** Calculated Interactions Energies for BTB Dimers.

Dimer	Electrostatic (Kcal/mol)	van der Waals (Kcal/mol)	Total (Kcal/mol)
Lola-Lola	-350	-204	-554
Lola-Rib	-386	-206	-592
Rib-Rib	-173	-201	-374

### Salivary gland polytene chromosome squashes

Third instar larval salivary glands were dissected in 1xPBS and fixed as follows: fix 1 (100 μl 37% formaldehyde, 700 μl H_2_O, 100 μl 10xPBS and 100 μl 10% Tween-20) for 1 minute, fix 2 (100 μl 37% formaldehyde, 300 μl H_2_O, 500 μl glacial acetic acid and 100 μl 10% Tween-20) for 2 minutes, fix 3 (550 μl H_2_O and 450 μl glacial acetic acid) for 5 minutes. After fixation the salivary glands were transferred onto a siliconized cover slip (using Sigmacote SL-2; Sigma-Aldrich), flipped over onto a poly-L-lysine treated slide, and squashed using the thumb to apply firm pressure onto the salivary glands for ~50–60 seconds. The slides were frozen in liquid nitrogen, the cover slip was popped off, and the slides were transferred to 1x PBS. The slides were washed 2 times for 30 minutes in PBST (1xPBS and 0.1% Tween-20) and 1 time for 30 minutes in antibody dilution buffer (1xPBS, 0.1% Triton-X-100, 5% milk). Slides were incubated overnight at 4°C in a humid chamber in antibody dilution buffer containing the following antibodies: mouse anti-H3K27me3 (1:125, Millipore); mouse anti-RNA Polymerase II phosphoserine 5 H14 (1:35, BioLegend), rat anti-HA (1:100, Roche Diagnostics), and/or rabbit anti-Lola (1:100, Edward Giniger [43]). Following incubation, slides were washed 3 times in PBST for 15 minutes and incubated for 1 hour at 37°C in the appropriate secondary antibodies, diluted 1:400 in antibody dilution buffer. After incubation, slides were washed once again 3 times in PBST for 15 minutes and mounted in DABCO for viewing. Polytene chromosomes were imaged on an Olympus Fluoview 1000 using the 488 and 561 lasers using a PlanApo N 60x oil (NA 1.42) objective. Images were processed using ImageJ software.

## Results

### Characterization of the role of Rib and Lola in gonad morphogenesis

Previously, *lola* and *rib* were identified in screens for genes that are required for embryonic gonad morphogenesis [37, 38]. In order to further characterize the gonad phenotypes of *rib* and *lola* mutants, the *68-77*-*lacZ* enhancer trap, which is expressed in the cytoplasm of the SGPs, was used to mark the cytoplasm of SGPs and examine gonad morphology (Fig 1C) [4, 5, 46]. During normal gonad development, SGP clusters fuse into an elongated, coalesced gonad by the end of stage 13, and PGCs and SGPs compact into a spherical gonad with SGPs ensheathing the PGCs by the end of stage 15 (Fig 1A and 1C). Therefore, we scored *rib* and *lola* embryonic gonads for a failure of SGP clusters to coalesce, referred to as fusion defects, and a failure of gonads to form a round spherical gonad, referred to as compaction defects. Immunostaining of SGPs of the gonad in both *rib* and *lola* mutants revealed defects in SGP cluster fusion and gonad compaction (Fig 1D–1H; S1 Fig). Quantification of these phenotypes demonstrates that the control embryos, which carry the *68-77-lacZ* enhancer trap, exhibit a low frequency of fusion and compaction defects of ~7% (Fig 1H). Less than 17% of the gonads in embryos heterozygous for *rib* and *lola* mutations exhibited fusion and compaction defects (Fig 1H). For analysis of *rib* and *lola* homozygous mutants, heteroallelic combinations were used to minimize the potential contribution of second site mutations to the observed phenotype, as these mutant alleles were obtained from a mutagenesis screen [37]. Four *lola* alleles were used in these studies: mutations in *lola*^*ORE76*^ and *lola*^*22*.*05*^ (Q97STOP) are predicted to affect all Lola protein isoforms [28, 38], while *lola*^*46*.*38*^ encodes a H844L mutation in the second the zinc finger of Lola isoform T (Flybase designation PR/PG) and *lola*^*ORC4*^ encodes a premature stop codon (P771STOP) in Lola isoform K (Flybase designation PI) (Fig 1B). We observed defects when each isoform-specific allele was combined with a null or hypomorphic allele affecting all isoforms (Fig 1D, 1E and 1H). In the case of the *lola*^*46*.*38/22*.*05*^ and *lola*^*22*.*05/ORE76*^ mutants, fusion and compaction defects were observed in just under 40% of gonads (Fig 1H). A small increase in the frequency of gonad defects to 44% was observed in *lola*^*46*.*38/ORE76*^ mutants (Fig 1H), suggesting that despite an early stop codon in *lola*^*22*.*05*^ it may not be as strong of a loss of function mutant as *lola*^*ORE76*^. *lola*^*22*.*05/ORC4*^ mutants carrying one copy of the Lola isoform K-specific mutation in combination with a mutation affecting the Lola common region exhibited gonad defects 34% of the time (Fig 1H). Thus, all heteroallelic *lola* mutants exhibited an increase in fusion and compaction defects when compared to their heterozygous counterparts. These results also suggest that multiple Lola isoforms, namely Lola-T and Lola-K, function in gonad morphogenesis.

In the case of the *rib* alleles, only the *rib*^*1*^ allele encodes a protein with a premature stop codon (R283STOP) [15], while *rib*^*35*.*14*^ encodes a protein with a D406V missense mutation, which is localized to the PSQ DNA binding motif and is likely to function as a hypomorph (Fig 1B). While the precise mutation in the *rib*^*55*.*25*^ allele is unknown, sequencing has revealed that it is not within the coding sequence. Examination of *rib*^*35*.*14/55*.*25*^ mutants reveals the presence of fusion and compaction defects in approximately 52% of embryonic gonads (Fig 1F–1H). *rib*^*55*.*25/1*^ heteroallelic mutants exhibited the highest frequency of defects with a total of 61% (Fig 1H). Thus, the *rib*^*55*.*25*^ mutant allele is likely a stronger allele than the *rib*^*35*.*14*^ allele. Overall, these results suggest that Rib and Lola are critical regulators of gonad coalescence and compaction.

Previous studies also demonstrated a requirement for Lola in germ cell migration, as germ cells were observed outside of the coalesced gonad [38]. Therefore, we scored the number of extragonadal germ cells in *rib* and *lola* mutant embryos. While controls had an average of less than one extragonadal germ cell per embryo, *rib* heterozygotes had a slight increase to an average of 1.5 extragonadal germ cells and homozygotes had a statistically significant increase to ~5 extragonadal germ cells per embryo (S2 Fig). In the case of *lola* mutants, heterozygotes had an average of ~5 extragonadal germ cells per embryo, while homozygotes had a statistically significant increase to ~10 extragonadal germ cells (S2 Fig). These results suggest that Rib and Lola also function to regulate germ cell migration cell autonomously or non-autonomously.

### Lola and Rib function in the SGPs to promote gonad development

**Fig 2:**
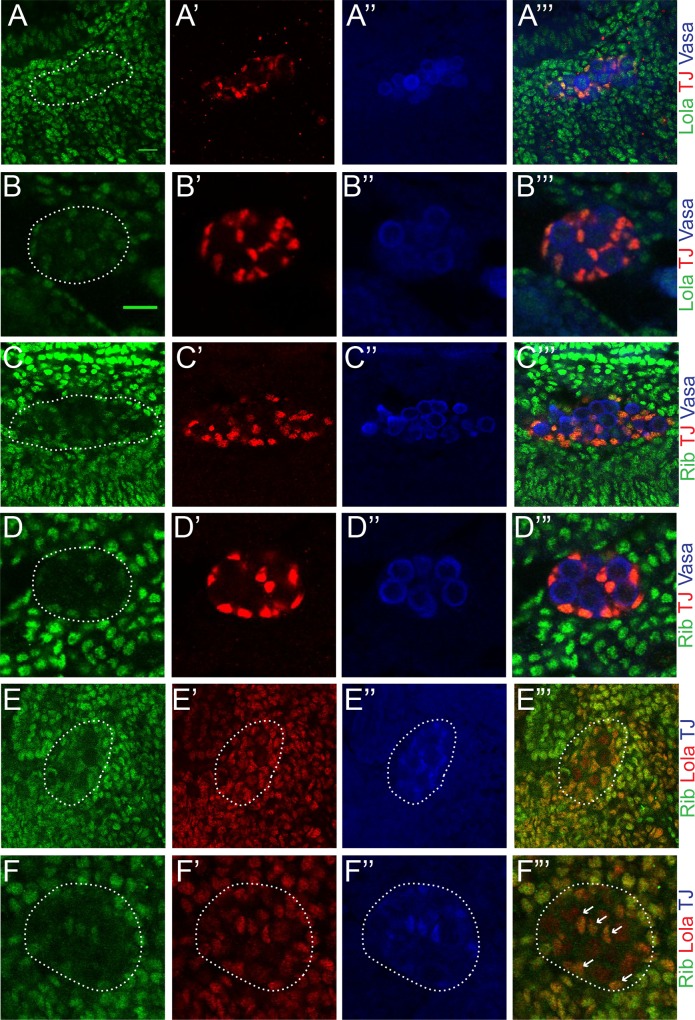
Expression of Rib and Lola in the embryonic gonad. (A-A”‘) Expression of Lola in an Oregon-R stage 13 gonad. (A) Anti-Lola (green). (A’) Anti-Traffic jam (TJ) marks somatic gonadal precursors (SGPs; red). (A”) Anti-Vasa marks primordial germ cells (PGCs; blue). (A”‘) Merged image with anti-Lola (green), anti-TJ (SGPs; red), and anti-Vasa (PGCs; blue). (B-B”‘) Expression of Lola in an Oregon-R stage 15 gonad. (B) Anti-Lola (green). (B’) Anti-TJ (SGPs; red). (B”) Anti-Vasa (PGCs; blue). (B”‘) Merged image with anti-Lola (green), anti-TJ (SGPs; red), and anti-Vasa (PGCs; blue). (C-C”‘) Expression of Rib in an Oregon-R stage 13 gonad. (C) Anti-Rib (green). (C’) Anti-TJ (SGPs; red). (C”) Anti-Vasa (PGCs; blue). (C”‘) Merged image with anti-Rib (green), anti-TJ (SGPs; red), anti-Vasa (PGCs; blue). Same scale as (A). (D-D”‘) Expression of Rib in an Oregon-R stage 15 gonad. Same scale as (B). (D) Anti-Rib (green). (D’) Anti-TJ (SGPs; red). (D”) Anti-Vasa (PGCs; blue). (D”‘) Merged image with anti-Rib (green), anti-TJ (SGPs; red), anti-Vasa (PGCs; blue). (E-E”‘) Colocalization of Rib and Lola in an Oregon-R stage 13 gonad. Same scale as (A). (E) Anti-Rib (green). (E’) Anti-Lola (red). (E”) Anti-TJ (SGPs; blue). (E”‘) Merge of anti-Rib (green) and anti-Lola (red). (F-F”‘) Colocalization of Rib and Lola in an Oregon-R stage 15 gonad. Same scale as (B). (F) Anti-Rib (green). (F’) Anti-Lola (red). (F”) Anti-TJ (SGPs; blue). (F”‘) Merge of anti-Rib (green) and anti-Lola (red). Gonads are outlined by dotted lines. Areas of high Rib-Lola colocalization are indicated by arrows. For all images posterior is to the right. Scale bars: 10μm.

The fusion and compaction defects observed in the gonad suggest that these proteins function in the SGPs during gonad morphogenesis, while germ cell migration defects suggest they may also be required in the PGCs. Therefore, immunohistochemistry for Rib and Lola proteins was performed to determine where Rib and Lola are expressed in the developing gonad. Antibody staining for Lola with an antibody that recognizes all Lola isoforms demonstrates that Lola is expressed in the SGPs and PGCs and the surrounding mesoderm in stage 13 embryos and and primarily in the SGPs and surrounding mesodermal cells in 15 embryos (Fig 2A and 2B). As our genetic analysis suggested Lola isoforms T and K may function in gonad morphogenesis, we also examined expression of these isoforms using Lola-T and Lola-K GFP fusion proteins. Lola-T is expressed in the SGPs and to a lesser extent the PGCs of stage 13 embryonic gonads, while it does not exhibit significant expression in Stage 15 gonads (S3A and S3B Fig). In contrast, Lola-K is expressed in the SGPs and PGCs of both stage 13 and stage 15 gonads (S3C and S3D Fig). These results support our earlier genetic analysis suggesting that both Lola-T and Lola-K function during gonad morphogenesis.

Antibody staining for Rib revealed expression of Rib in the SGPs and no staining in the PGCs in stage 13 and 15 embryos (Fig 2C and 2D). Given the similar phenotypes observed in *rib* and *lola* mutants and similar expression patterns in the SGPs of the embryonic gonad, the colocalization of both of these proteins was examined. Immunohistochemistry analysis reveals the colocalization of Lola and Rib in SGPs in stage 13 and 15 embryos (Fig 2E and 2F), suggesting that Lola and Rib are both required in the SGPs to promote gonad morphogenesis. Rescue experiments previously demonstrated that overexpression of Lola in the mesoderm could rescue *lola* mutant gonad phenotypes [38]. Therefore, the ability of Rib overexpression in the mesoderm to rescue the *rib* mutant phenotype was tested. Results demonstrate that Rib overexpression in the mesoderm rescues the *rib* mutant phenotype (S4 Fig).

**Fig 3:**
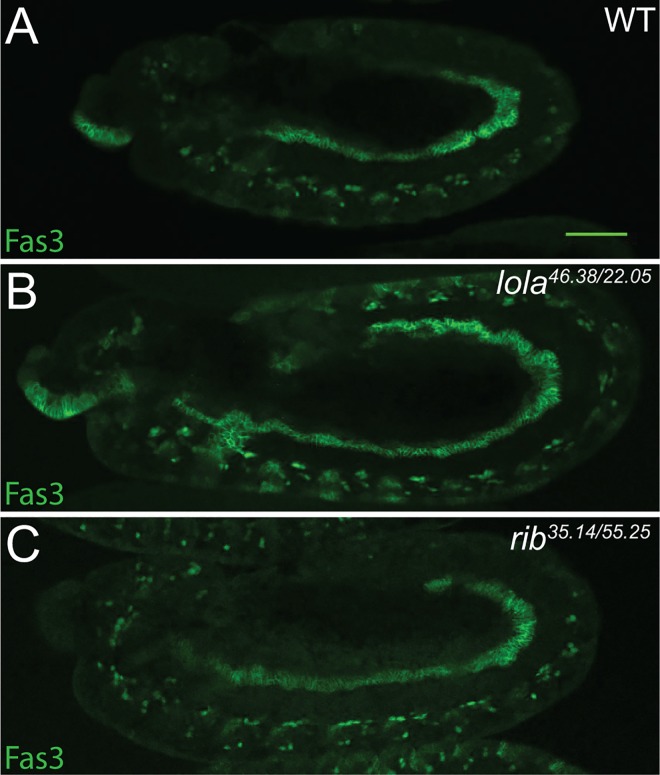
Mesoderm develops normally in *rib* and *lola* mutants. (A) Oregon-R wild-type (WT) control (n = 10), (B) *lola*^*46*.*38/22*.*05*^ (n = 9), *and* (C) *rib*^*35*.*14/55*.*25*^ (n = 5) stage 12 embryos immunostained for the visceral mesodermal marker Fasciclin 3. For all images posterior is to the right. Scale bar: 50 μm.

Because *lola* and *rib* expression is also observed in the surrounding mesoderm, it was necessary to eliminate the possibility that the gonad defects were due to a more generalized defect in mesoderm development. In order to look at mesoderm development, stage 12 embryos were immunostained with anti-Fasciclin 3 to examine visceral mesoderm development (Fig 3). Visceral mesoderm development was indistinguishable from controls for both *lola* and *rib* mutant embryos (Fig 3), demonstrating that mesoderm development was not globally disrupted in *rib* and *lola* mutants. Thus, expression data suggests Rib and Lola function specifically in the SGPs to promote gonad morphogenesis.

### *rib* and *lola* genetically interact

**Fig 4:**
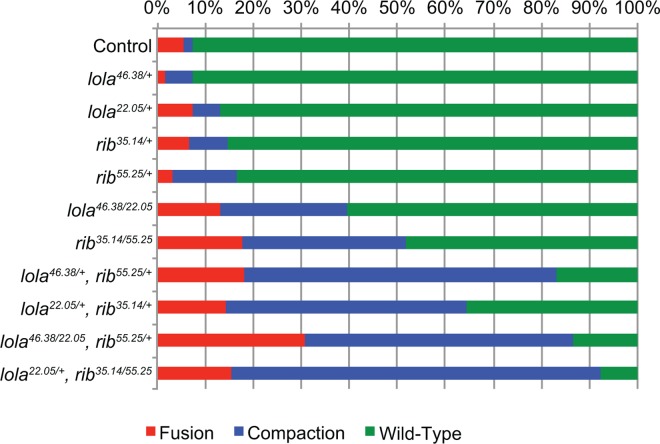
*rib and lola* genetically interact. Graph of phenotypic frequency for stage 15 embryonic gonads. The following gonad phenotypes were scored: fusion (red), compaction (blue) and wild-type (green). Gonads were scored by staining somatic gonadal precursor cells for the *68-77-lacZ* enhancer trap. A chi-square test was performed to test the null hypothesis that the phenotype ratios would be the same across all genotypes. Results allow us to reject the null hypothesis: *Χ*^2^_*22*, *0*.*05*_ = 313.31, p<0.001.

With the observations that both *rib* and *lola* exhibit similar mutant defects and are both expressed in the SGPs, we explored the possibility these proteins may cooperate to regulate gonad morphogenesis through genetic interaction studies. Gonad development was examined in embryos heterozygous for both *rib* and *lola* alleles. Stage 15 embryonic gonads were scored as wild-type, or as having fusion or compaction defects. The frequency of defects in double heterozygotes was compared to control embryos, as well as to embryos heterozygous for either *rib* or *lola* (*lola*^*46*.*38/+*^, *rib*^*35*.*14/+*^, *lola*^*22*.*05/+*^, and *rib*^*55*.*25/+*^) (Fig 4). The percentage of defective gonads ranged from 8–17% in the controls (Fig 4). In the case of embryos heterozygous for both *rib* and *lola* (*lola*^*46*.*38/+*^, *rib*^*55*.*25/+*^ and *lola*^*22*.*05/+*^, *rib*^*35*.*14/+*^), the frequency of gonad defects increased significantly to between 65–85% (Fig 4), demonstrating a synergistic effect and suggesting that *rib* and *lola* cooperate to regulate gonad morphogenesis. In addition, heteroallelic mutants for *rib* or *lola* were examined that were also heterozygous for *lola* or *rib*, respectively (*lola*^*22*.*05/+*^, *rib*^*35*.*14/55*.*25*^ and *lola*^*46*.*38/22*.*05*^, *rib*^*55*.*25/+*^). These mutants showed a dramatic increase in gonad defects relative to *rib* and *lola* mutants alone with more than 85% of the gonads exhibiting fusion and compaction defects, compared to ~50% in *rib* and ~40% in *lola* heteroallelic combinations (Fig 4). These results suggest that Rib and Lola function cooperatively in the same pathway or in parallel pathways to regulate embryonic gonad development.

### Rib and Lola do not regulate expression of each other

**Fig 5:**
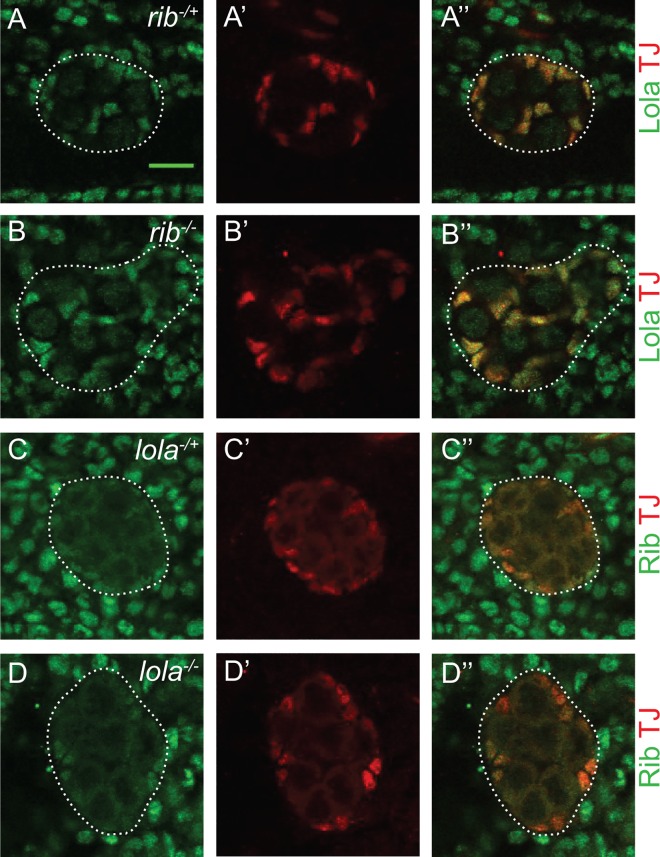
Lola and Rib do not regulate expression of each other. (A-B”) Lola expression in *rib* heterozygous and homozygous mutant stage 15 gonads, posterior to the right. Anti-Lola (green) and anti-Traffic jam (TJ) marks SGPs (red). (A-A”) *rib*^*+/-*^ gonad (*rib*^*35*.*14/+*^ or *rib*^*55*.*25/+*^) (n = 16). (B-B”) *rib*^*35*.*14/55*.*25*^ gonad (n = 11). (C-D”) Rib expression in *lola* heterozygous and homozygous mutant stage 15 gonads, posterior to the right. Anti-Rib (green) and anti-TJ marks SGPs (red). (C-C”) *lola*^*+/-*^ gonad (*lola*^*46*.*38/+*^ or *lola*^*22*.*05/+*^) (n = 11). (D-D”) *lola*^*46*.*38/22*.*05*^ gonad (n = 11). The gonad is outlined with a dotted line. Scale bar: 10μm.

The genetic interaction of *rib* and *lola* led us to hypothesize that Rib and Lola could function (1) in a stepwise fashion in a single pathway; (2) in parallel pathways to impinge on a common set of downstream targets; or (3) as part of a complex to regulate a common set of target genes to promote gonad morphogenesis. Therefore, we first explored the possibility that Rib and Lola regulate expression of one another in a stepwise fashion by examining expression of Lola in *rib* mutants and expression of Rib in *lola* mutants. Expression of Lola in *rib* heterozygotes versus *rib* heteroallelic mutants revealed no difference in Lola expression, suggesting that Rib does not regulate Lola expression levels (Fig 5A and 5B). Similarly, Rib expression was compared in *lola* heterozygotes and *lola* heteroallelic mutants, and Rib expression was unchanged (Fig 5C and 5D). These results suggest that Rib and Lola are not regulating expression of each other, but rather function in parallel pathways or as a complex to regulate gene expression during embryonic gonad development.

### Rib and Lola physically interact via their BTB domains

**Fig 6:**
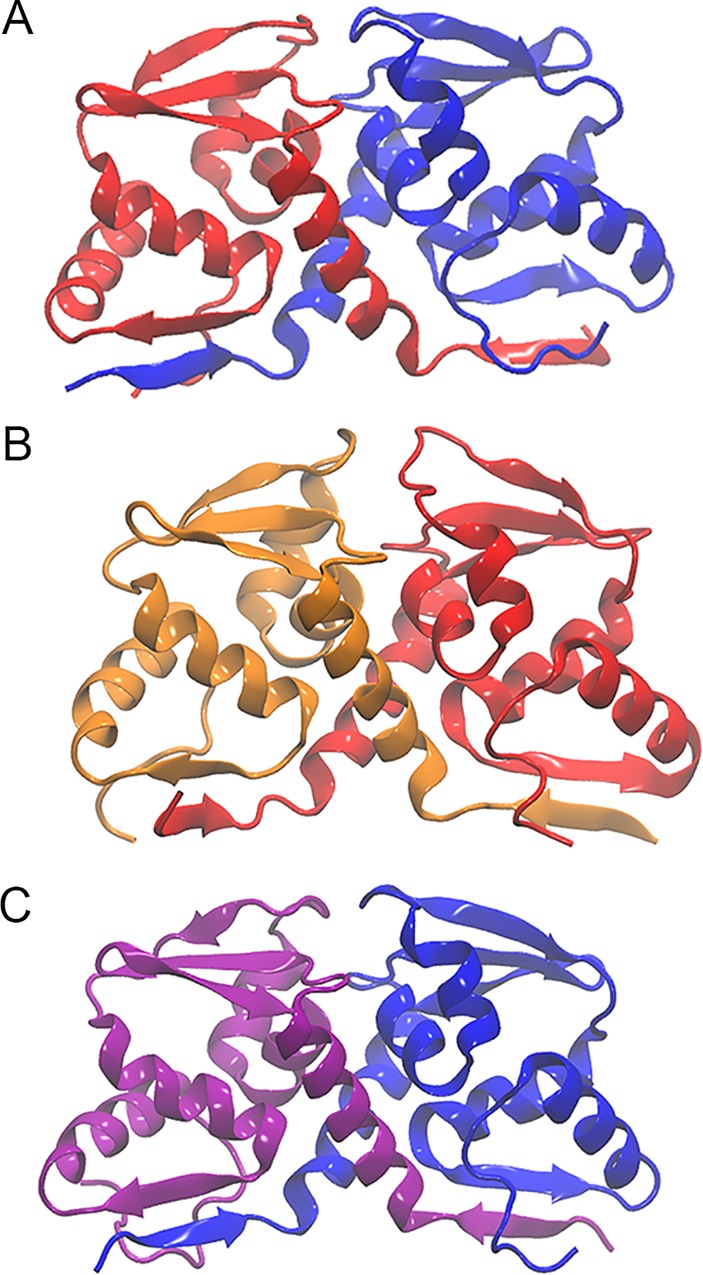
Protein modeling of Rib and Lola BTB domain interactions predicts heterodimerization and homodimerization. (A) Lola (red)-Rib (blue) BTB domain heterodimer. (B) Lola-Lola BTB domain homodimer. (C) Rib-Rib BTB domain homodimer.

Given the observations that *rib* and *lola* show a strong genetic interaction, co-localize in the SGPs, and both contain a BTB domain, we next tested the possibility that these proteins may physically interact to regulate embryonic gonad development. Homology modeling and molecular dynamics simulations were used to predict the potential interaction between Lola and Rib BTB domains (Fig 6). Interaction energy calculations (Table 1) suggest that Rib and Lola have the ability to heterodimerize as well as homodimerize.

**Fig 7:**
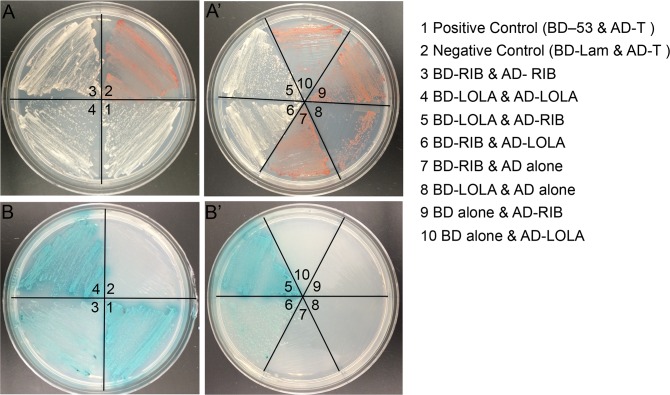
Rib and Lola physically interact via their BTB domains by yeast two-hybrid analysis. (A-A’) Growth on SD-Leu-Trp plates illustrates successful yeast mating. (B-B’) SD-Leu-Trp-His-Ade plates with X-α-gal and Aureobasidin A were used to test for interaction of activation domain (AD) and DNA binding domain (BD) fusion proteins.

Therefore, we tested the ability of the Lola and Rib BTB domains to interact physically by performing a yeast two-hybrid assay. The Rib and Lola BTB domains were each fused to the yeast GAL4 DNA binding domain (BD) and the GAL4 activation domain (AD). Positive and negative controls, as well as strains expressing BD-RIB and AD-Lola, AD-Rib and BD-Lola, AD-Rib and BD-Rib, and AD-Lola and BD-Lola were successfully mated and found to be viable when carrying all fusion protein combinations (Fig 7A and 7A’). The ability of AD and BD fusion proteins to interact were tested using four reporters under the control of the GAL4 upstream activating sequence: His3, Ade2, *LacZ*, and the *AUR1-C* gene, which confers resistance to Aureobasidin A (Fig 7B and 7B’). The positive control (Fig 7B #1) exhibited activation of all reporters, while the negative control (Fig 7B #2) failed to grow on the selection plates. Lola-BD and Rib-BD fusions were tested with the GAL4-AD alone and failed to activate reporters (Fig 7B’ #7–8), demonstrating that Rib and Lola BTB domains do not autoactivate reporters. Additionally, Lola-AD and Rib-AD fusions were also tested in combination with the GAL4-BD alone and failed to activate reporters (Fig 7B’ #9–10), demonstrating that the Lola and Rib BTB domains do not bind to the reporters or the GAL4-BD nonspecifically. Mating of BD-Lola with AD-Rib and BD-Rib with AD-Lola resulted in activation of all reporters (Fig 7B’ #5–6). In addition, we tested the ability of the Rib and Lola BTB domains to form homodimers, and observed homodimerization of both Rib and Lola BTB domains, with a more robust interaction for Lola than for Rib (Fig 7B #3–4). These results are consistent with molecular modeling, which predicted stronger interactions for Lola-Lola homodimers than for Rib-Rib homodimers (Table 1). These results demonstrate that Rib and Lola BTB domains are capable of homo- and heterodimerization, and suggest that Rib and Lola form a complex to cooperatively regulate gene expression during gonad morphogenesis rather than simply functioning in parallel.

### Rib and Lola are transcriptional activators and repressors

**Fig 8:**
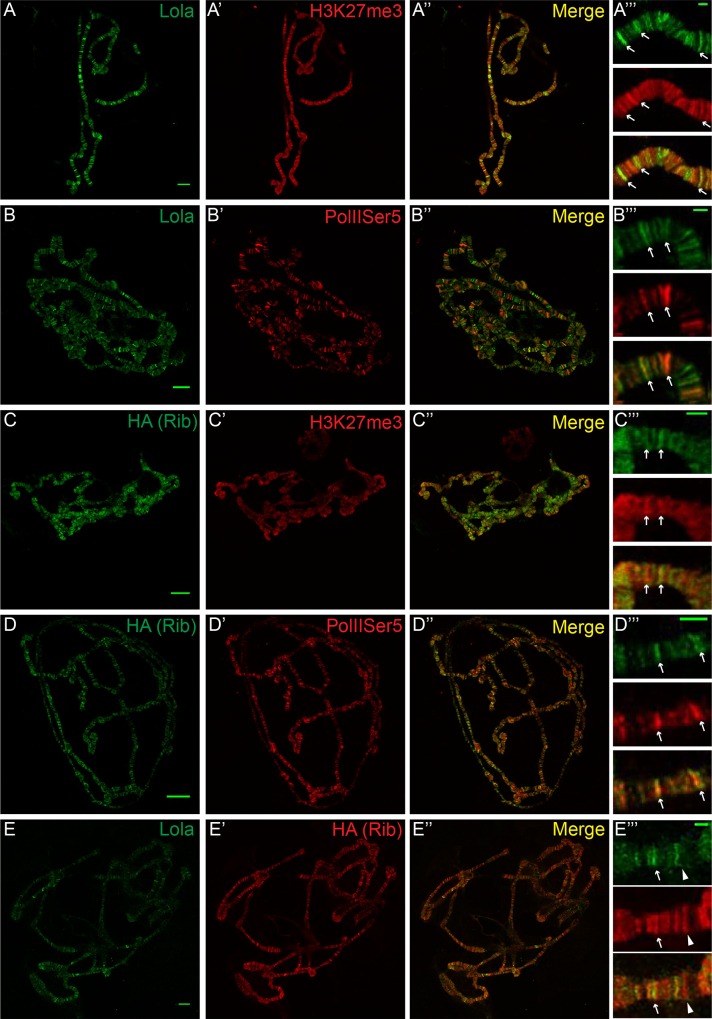
Colocalization of Rib and Lola with marks of transcriptional activation and repression. Immunofluorescence staining of polytene chromosomes of third instar larval salivary gland. (A-A”‘) Oregon-R polytene chromosomes stained with anti-Lola (green) and anti-H3K27me3 (red), with the merge showing areas of colocalization. (A”‘) Zoomed images of (A-A”). (B-B”‘) Oregon-R polytene chromosomes stained with anti-Lola (green) and anti-PolIIser5 (red), with the merge showing areas of colocalization. (B”‘) Zoomed images of (B-B”). (C-C”‘) *forkhead*-Gal4; UAS-3xHA-Rib polytene chromosomes stained with anti-HA (Rib; green) and anti-H3K27me3 (red), with the merge showing areas of colocalization. (C”‘) Zoomed images of (C-C”). (D-D”‘) *forkhead*-Gal4; UAS-3xHA-Rib polytene chromosomes stained with anti-HA (Rib; green) and anti-PolIIser5 (red), with the merge showing areas of colocalization. (D”‘) Zoomed images of (D-D”). (E-E”‘) *forkhead*-Gal4; UAS-3xHA-Rib polytene chromosomes stained with anti-Lola (green) and anti-HA (Rib; red), with the merge showing areas of colocalization. (E”‘) Zoomed images of (E-E”). Arrowheads indicate region with Rib lacking Lola. Scale bars in unzoomed images: 10μm. Scale bars in zoomed images: 2μm. Arrows indicate colocalization. For each experiment a minimum of 10 polytene chromosomes were examined.

While earlier studies suggested that Rib and Lola function as transcriptional repressors, more recently it has been suggested that Rib and Lola also function in transcriptional activation [19, 36, 42]. In order to further examine the roles of Rib and Lola as transcriptional activators and/or repressors, we examined the localization of the Rib and Lola protein with marks of transcriptional activation and repression on polytene chromosomes. First, Rib and Lola expression were examined in combination with immunostaining for H3K27me3, a mark of transcriptional repression. Results demonstrated that Lola colocalized with H3K27me3 on numerous sites on the chromosome (Fig 8A). As the Rib antibody did not exhibit strong staining on polytene chromosomes, we expressed a 3xHA-tagged Rib in salivary glands using *forkhead*-Gal4. We observed colocalization of 3xHA-Rib and H3K27me3 at numerous sites on polytene chromosomes (Fig 8C). While Lola and Rib colocalize with H3K27me3 at a number of loci on polytene chromosomes, the presence of Lola and Rib at other distinct sites suggests that Rib and Lola may also localize at sites of transcriptional activation. In order to determine if Lola and Rib may function in transcriptional activation, we examined Rib and Lola colocalization with sites of RNA Polymerase II phosphoserine 5 (PolIIser5). Lola and Rib also colocalized with PolIIser5 at many sites on polytene chromsomes (Fig 8B and 8D). These results suggest that Rib and Lola have dual functions as transcriptional activators and repressors. As our earlier results suggest that Rib and Lola may coregulate transcription of target genes, we examined colocalization of 3xHA-Rib and Lola on polytene chromosomes. While Lola and Rib colocalize at some loci, Lola staining on polytene chromosomes is significantly reduced upon overexpression of 3xHA-Rib, and there are a number of loci to which only Rib localizes (Fig 8E). These results suggest that Rib does not require Lola to localize to polytene chromosomes. In addition, as Lola staining was present at more sites on the polytene chromosomes when endogenous Rib was not observed, it does not appear that Rib is required to recruit Lola. The reduction of Lola staining on chromosomes upon overexpression of 3xHA-Rib suggests that the 3xHA tag may affect the ability of Rib to dimerize or that overexpressed Rib may result in an increased population of Rib homodimers at the expense of Rib-Lola heterodimers. The continued colocalization of Rib and Lola at some sites suggests that Rib and Lola cooperate to regulate transcription of a subset of target genes, while functioning independently of each other to regulate other targets.

### Expression analysis of Rib and Lola transcriptional targets

Previous studies have resulted in the identification of a number of Rib and Lola downstream targets in other tissues. Expression profiling and genetic analysis has demonstrated that Lola promotes expression of Slit and Robo in the developing central nervous system and inhibits expression of the cytoskeletal regulator Spire (Spir). Both Slit and Robo have been demonstrated to function in gonad morphogenesis; however, only Robo has been localized to the gonad by immunostaining [37]. Therefore, we examined expression of Robo and Spir in *lola* null homozygotes, and compared expression to *lola* heterozygotes. Immunostaining for Robo revealed that mutation of *lola* does not alter Robo expression in the gonad (S5A and S5B Fig). These results are consistent with previous observations that Lola does not regulate Robo expression in the gonad [38]. Next we examined expression of Spir in the gonad. Antibody staining reveals little enrichment of Spir in the developing gonad, and its expression does not appear to be altered in *Lola* homozygotes, as compared to heterozygous controls (S5C and S5D Fig). These results suggest that Lola regulates different targets in a tissue-specific manner.

In the case of Rib, chromatin immunoprecipitation and gene expression analysis have resulted in the identification of more than a dozen Rib transcriptional targets in the salivary gland, including *crb*, which encodes an apical membrane protein involved in epithelial polarity. In addition, Moesin (Moe), the sole Ezrin-Radixin-Moesin protein in *Drosophila*, was also found to exhibit increased phosphorylation upon *rib* mutation. Therefore, expression of *crb* and activation phospho-Moesin (pMoe) were examined in *rib* mutant embryos. Analysis of *crb* expression in the gonad reveals that *crb* is not highly expressed in the developing gonad, and its expression levels do not change in *rib* homozygotes relative to heterozygous controls (S6A and S6B Fig). While pMoe is present in the gonad at the PGC membrane, it does not appear in the SGPs and its expression also remains unchanged upon *rib* mutation (S6C and S6D Fig). These results suggest that Rib functions through different downstream targets in the gonad as compared to the salivary gland.

## Discussion

In this study we demonstrated the requirement for two BTB family transcription factors, Rib and Lola, for embryonic gonad morphogenesis. Both *rib* and *lola* are expressed in the SGPs of the gonad, and *rib* and *lola* mutants exhibit defects in SGP cluster fusion and gonad compaction. Genetic analysis revealed that these genes interact with each other in the context of gonad development, while molecular modeling and yeast two-hybrid studies demonstrate the ability of these proteins to interact physically via their BTB domains. We show that Rib and Lola colocalize with marks of transcriptional activation and repression, suggesting that these proteins act as dual-function transcriptional regulators to promote tissue morphogenesis. Finally, we find that Rib and Lola do not regulate the same downstream targets in the gonad as those previously identified in other developmental contexts.

Rib and Lola were identified in screens for mutations that affect embryonic gonad morphogenesis [37, 38]. The defects in SGP cluster fusion and gonad compaction that we and others have observed, as well as previous work demonstrating SGP cluster fusion and compaction do not require PGCs, suggest that Rib and Lola function specifically in the SGPs to regulate gonad development [1, 37, 38, 64]. Consistent with the mutant phenotypes, both proteins are strongly expressed in the SGPs; however, they are also expressed strongly in the surrounding mesoderm and in the case of Lola, weakly in the PGCs (Fig 2). The proper specification of other mesodermally derived tissues, like the visceral mesoderm (Fig 4), suggests that the gonad defects observed in *rib* and *lola* mutants are due to a specific requirement for these proteins in the SGPs. Supporting this hypothesis, previous work demonstrated that overexpression of *lola* in the mesoderm can rescue the *lola* mutant phenotype [38], and we find that overexpression of Rib in the mesoderm can rescue the *rib* mutant phenotype (S4 Fig). Interestingly, extragonadal PGCs were also observed in many *rib* and *lola* mutant embryos (S2 Fig), suggesting that Rib and Lola may regulate the migration of PGCs and/or their ability to interact with SGPs cell autonomously or non-autonomously. As Rib expression is not observed in PGCs (Fig 2C and 2D), and Lola rescue experiments indicated that Lola mesodermal expression is sufficient to rescue the *lola* mutant phenotype [38], we favor the hypothesis that regulation of PGC migration by Rib and Lola must be cell non-autonomous.

Identification of the downstream targets through which Rib and Lola function to regulate cell-cell interactions is critical for understanding how these proteins regulate tissue morphogenesis. Both Rib and Lola contain DNA binding domains, with Rib containing a PSQ motif, and many Lola isoforms containing at least one zinc finger domain. Earlier studies predicted Lola and Rib to function as transcriptional regulators [15, 16, 28], which has subsequently been confirmed [19, 36]. In the case of Lola, numerous putative targets of Lola have been identified in the central nervous system, including the cytoskeletal regulator Spir, as well as members of the Slit/Robo signaling pathway [33, 43]. Consistent with a previous study, we observe that *robo* expression does not appear to be regulated by Lola in the gonad [37, 38] (S5A and S5B Fig). In addition, our results also demonstrate that Lola does not regulate *spir* expression to promote gonad morphogenesis (S5C and S5D Fig). These results suggest that Lola is likely to regulate different target genes in different tissues. In the case of the only confirmed direct transcriptional target of Lola, the *copia* retrotransposon, Lola appears to repress *copia* expression in the central nervous system, while activating its expression *copia* in the gonad [19]. These results suggesting that a single Lola isoform may have dual functions or that different Lola isoforms regulate *copia* expression in different tissues. The ability of Lola isoforms to function as transcriptional activators and repressors is supported by the localization of Lola with both active and repressive marks of transcription on polytene chromosomes (Fig 8A and 8B).

The existence of at least 20 different Lola protein isoforms, 17 of which contain one or two zinc fingers that lack sequence conservation and exhibit different binding specificities, suggests that the different Lola isoforms regulate distinct cohorts of genes and may function in a tissue-specific manner [22, 27–30]. Consistent with this prediction, we and others have found that different isoforms are expressed and function in different tissues [22, 28, 38]. In the embryo we find that Lola-T, which is specifically mutated by the *lola*^*46*.*38*^ allele, is required for gonad morphogenesis, consistent with a previous study (Fig 1) [38]. However, we also observe defects in gonad morphogenesis with a Lola-K isoform-specific mutant allele, *lola*^*ORC4*^ (Fig 1). Comparison of the T and K isoforms reveals significant sequence similarity in their zinc finger regions [28], as well as similarity in their predicted DNA consensus binding sequences [30], suggesting that these isoforms may regulate transcription of a common set of target genes. Expression analysis of Lola-K and Lola-T GFP fusion proteins in the gonad revealed that Lola-T is expressed in SGPs in stage 13 gonads, while Lola-K-GFP was strongly expressed in the SGPs and PGCs of both stage 13 and 15 gonads (S3 Fig). These results suggest that both Lola-T and Lola-K function in the SGPs to regulate gonad morphogenesis. Expression of Lola-K in PGCs suggests that this isoform may be specifically required in the PGCs. Interestingly, it was previously reported that overexpression of *lola-T* rescues the *lola* mutant gonad phenotype in mutants predicted to lack all Lola isoforms [38]. This data suggests that different isoforms may be able to compensate for each other if they share DNA binding similarities, as is the case for Lola-K and Lola-T. It is also feasible that different Lola isoforms dimerize to cooperatively regulate gonad development, which is supported by our molecular modeling and yeast-two hybrid analysis demonstrating the ability of *Drosophila* BTB domains to homodimerize (Figs 6 and 7) [31, 32].

The transcriptional activity of Lola could also depend on its interaction with other BTB and/or non-BTB domain-containing proteins, including Rib. *Drosophila* BTB domain-containing proteins have been demonstrated to interact with each other in yeast-two hybrid analysis [18]. In addition, BTB containing proteins, including Pipsqueak, Trithorax-like, Batman and Bric-à-brac, function together to limit ovariole number in the *Drosophila* ovary [65]. In the context of the salivary gland and the trachea, Rib interacts with another BTB family protein, Lolal, to regulate development [42]. Molecular modeling of Lola-Rib BTB domain interactions (Fig 6), suggested that Lola and Rib BTB domains are capable of forming heterodimers, as well as homodimers. This hypothesis was supported by genetic interaction analysis of *rib* and *lola* mutants and the physical interaction of their BTB domains (Figs 4 and 7). Colocalization of Rib and Lola on polytene chromosomes reveals that Rib and Lola colocalize at some, but not all sites on the chromosomes (Fig 8E). This data combined with molecular modeling results suggests that Rib and Lola homodimers and heterodimers are likely to regulate transcription of different cohorts of genes to promote gonad morphogenesis.

Our understanding of how Rib functions to regulate tissue morphogenesis comes from work examining its role in salivary gland and tracheal development. Previous studies revealed that *rib* mutants exhibit defects in tube elongation that arise from decreased apical membrane length and increased apical stiffness [15, 16, 41]. Consistent with these phenotypes, *rib* mutants exhibit changes in apically-localized proteins, including decreased expression of *crb*, increased levels of active of Moe, and decreased levels of the recycling endosomal protein Rab11 [42]. Further examination of the basis of the *rib* mutant phenotype showed that these defects arise from a failure of *rib* mutants to execute the cell shape and volume changes needed for salivary gland morphogenesis [36]. Recent Rib ChIP-seq analysis and expression profiling of *rib* mutants resulted in the identification of 20 potential targets for transcriptional activation and 40 potential targets for transcriptional repression in the salivary gland [36], including genes involved in cell migration, cell adhesion, and regulation of the cytoskeleton [36]. Similar to the salivary gland and trachea, gonad morphogenesis depends on changes in SGP cell shape [64], suggesting that Rib may function through some of the same downstream targets to promote proper gonad formation. We have examined the ability of Rib to regulate expression of *crb* and phosphorylation of Moe. In the case of Crb, low levels of Crb protein are observed in the gonad, unlike the strong membrane expression observed in the salivary gland (S6A Fig), which is not surprising giving that neither SGPs or PGCs have been observed to exhibit polarity. This expression is unchanged in *rib* homozygotes, demonstrating that *crb* does not appear to be a target of Rib in the gonad (S6A and S6B Fig). While pMoe is strongly enriched at the PGC membrane, levels of pMoe remain unchanged in *rib* homozygotes (Fig 6C and 6D). Therefore, it appears that Rib regulates different downstream targets in a tissue-specific manner. This activity may depend on its interaction partners, as Rib interacts with different binding partners in different tissues, namely Lolal in the salivary gland and trachea and with Lola in the gonad [42].

In addition to identifying the downstream targets of Rib and Lola in the developing gonad, it is also critical to characterize the molecular mechanisms regulating Rib and Lola expression and activity. Rib has been suggested to function downstream of the Mitogen Activated Protein Kinase (MAPK) signaling pathway based on the similarity of mutant phenotypes of *rib* and members of the Fibroblast Growth Factor-MAPK signaling pathway, as well as the presence of seven MAPK consensus phosphorylation sites in Rib [16]. In contrast, the mechanisms regulating Lola’s functions are unknown. Identification of signaling pathways with which *rib* and *lola* genetically interact would provide insight into the mechanisms regulating expression and function of these proteins and allow us to better understand how they are functioning within a network to regulate tissue development.

Although there are no human orthologs of *lola* and *rib*, there are similar proteins to Lola in vertebrates, which include Zfp131, Miz-1, and Leukemia-Related Factor (LRF) [66]. Zfp131 exhibits the most similar expression pattern to Lola, as it is expressed in the testes, adult brain and the developing nervous system [66, 67]. In contrast, Rib does not have any similar vertebrate proteins, as the BTB domain has not been observed in combination with the PSQ motif in vertebrates. However, other BTB family proteins may functionally substitute for Rib in the context of vertebrate gonad development. The characterization of the roles of Rib and Lola in embryonic gonad development, their genetic and physical interaction, and their colocalization with regions of active and inactive transcription on polytenes chromosomes suggests that cooperative regulation of gene expression by BTB family proteins may be used in a variety of developmental and disease contexts. The implication of *Drosophila* and mammalian BTB proteins in lymphocyte, skeletal, gonad and neurological development, as well as in cancer, illustrates the importance of understanding the mechanisms by which these proteins cooperate to regulate gene expression [21]. Identification of the direct downstream transcriptional targets of these genes and the molecular pathways in which they function is critical for understanding how these genes regulate development and contribute to disease.

## Supporting Information

S1 Fig *lola* and *rib* mutants exhibit defects in gonad morphogenesis. (A-A”‘) Control stage 15 embryo expressing the *68-77-lacZ* enhancer trap. SGPs are labeled by anti-β-galactosidase (βgal; green); anti-Neurotactin (Nrt) labels the cell surface of somatic cells (red); and anti-Vasa labels the primordial germ cells (PGCs; blue). Arrows indicate SGP extensions ensheathing the PGCs. Scale bar: 10μm. (B-B”‘) *lola*^*46*.*38/22*.*05*^ stage 15 embryonic gonad exhibiting compaction defect. Cells are labeled with anti-βgal (SGPs; green), anti-Nrt (red), and anti-Vasa (PGCs; blue). Arrows indicate SGP extensions ensheathing the PGCs. (C-C”‘) *rib*^*35*.*14/55*.*25*^ stage 15 embryonic gonad exhibiting fusion defect. Cells are labeled with anti-βgal (SGPs; green), anti-Nrt (red), and anti-Vasa (PGCs; blue). Arrows indicate SGP extensions ensheathing the PGCs. All images are to the same scale and are a Z-projection from a stack of confocal images.
(TIF)

S2 Fig Mutation of *rib* and *lola* results in extragonadal germ cells. Stage 14 and 15 embryos were scored for the number of germ cells that fail to coalesce with SGPs during gonad morphogenesis. Embryos were immunostained with anti-Vasa to label the primordial germ cells and anti-GFP for genotyping. Representative embryos from the following genotypes are shown: (A) Control (*68-77-lacZ*), (B) *rib*^*+/-*^ (*rib*^*55*.*25/+*^ or *rib*^*35*.*14/+*^), (C) *rib*^*-/-*^ (*rib*^*55*.*25/35*.*14*^), (D) *lola*^*+/-*^ (*lola*^*46*.*38/+*^ or *lola*^*22*.*05/+*^), and (E) *lola*^*-/-*^ (*lola*^*46*.*38/22*.*05*^). (F) Quantification of the average number of extragonadal germ cells in the genotypes described above. A two-tailed, unpaired student t-test was performed to test the significance and results are noted as follows: * = p<0.01; ** = p0.001.
(TIF)

S3 Fig Expression of Lola isoforms in the embryonic gonad. (A-A”) Expression of Lola-T in a stage 13 gonad. Image is representative of 6/6 gonads. (A) Lola-T (anti-GFP; green). (A’) Anti-Eyes absent (Eya) marks somatic gonadal precursors (SGPs; red). (A”) Merged image with anti-Lola (anti-GFP; green), anti-Eya (SGPs; red), and anti-Vasa (PGCs; blue). (B-B”) Expression of Lola-T in a stage 15 gonad. Image is representative of 11/11 gonads. (B) Lola-T (anti-GFP; green). (B’) Anti-Eya (SGPs; red). (B”) Merged image with anti-Lola (anti-GFP; green), anti-Eya (SGPs; red), and anti-Vasa (PGCs; blue). (C-C”) Expression of Lola-K in a stage 13 gonad. (C) Lola-K (anti-GFP; green). Image is representative of 13/13 gonads. (C’) Anti- Eya (SGPs; red). (C”) Merged image with anti-Lola (anti-GFP; green), anti-Eya (SGPs; red), and anti-Vasa (PGCs; blue). (D-D”) Expression of Lola-T in a stage 15 gonad. (D) Lola-T (anti-GFP; green). (D’) Anti-Eyes absent (EYA) marks somatic gonadal precursors (SGPs; red). Image is representative of 9/9 gonads. (D”) Merged image with anti-Lola (anti-GFP; green), anti-EYA (SGPs; red), and anti-Vasa (PGCs; blue). The gonad is outlined with a dotted line.
(TIF)

S4 Fig Overexpression of *rib* in the mesoderm rescues the *rib* mutant phenotype. Graph of phenotypic frequency for stage 15 embryonic gonads. The following gonad phenotypes were scored: fusion (red), compaction (blue) and wild-type (green). Gonads were scored by staining somatic gonadal precursor cells for the *68-77-lacZ* enhancer trap. A Chi-square test was performed to test the null hypothesis that the phenotype ratios will be the same for all genotypes. Results allow us to reject the null hypothesis: *Χ*^2^_*4*, *0*.*05*_ = 29.836, p<0.001.
(TIF)

S5 Fig Lola does not regulate Robo or Spir in the gonad. (A-B”‘) Roundabout (Robo) expression in *lola* heterozygous and homozygous mutant stage 15 gonads, posterior to the right. Anti-Traffic Jam (TJ) marks the SGPs (green); anti-Robo (red) and anti-Vasa marks the PGCs (blue). (A-A”‘) *lola*^*ORE76/+*^ gonad. Nine stage 15/16 gonads were examined and a representative result is shown. (B-B”‘) *lola*^*ORE76/ORE76*^ gonad. Ten stage 15/16 gonads were examined and a representative result is shown. (C-D”‘) Spire (Spir) expression in *lola* heterozygous and homozygous mutant stage 15 gonads, posterior to the right. Anti-TJ (SGPs; green); anti-Spir (red), and anti-Vasa (PGCs; blue). (C-C”‘) *lola*^*ORE76/+*^ gonad. Seventeen stage 14/15 gonads were examined and a representative result is shown. (D-D”‘) *lola*^*ORE76/ ORE76*^ gonad. Eighteen stage 14/15 gonads were examined and a representative result is shown. Settings on the confocal microscope were held constant for detection of Robo and Spir in all images. All images are to the same scale. Scale bar: 10μm.
(TIF)

S6 Fig Rib does not regulate Crb expression or Moe activation in the gonad. (A-B”‘) Crumbs (Crb) expression in *rib* heterozygous and homozygous mutant stage 15 gonads, posterior to the right. Anti-β-galactosidase (βgal) marks the SGPs due to the presence of the *68-77-lacZ* enhancer trap (green); anti-Crb (red) and anti-Vasa marks the PGCs (blue). (A-A”‘) *rib*^*+/-*^ gonad (*rib*^*35*.*14/+*^ or *rib*^*55*.*25/+*^). Twenty-one stage 14/15 gonads were examined and a representative result is shown. (B-B”) *rib*^*35*.*14/55*.*25*^ gonad. Thirty-one stage 14/15 gonads were examined and a representative result is shown. (C-D”‘) Phospho-Moesin (pMoe) levels in *rib* heterozygous and homozygous mutant stage 15 gonads, posterior to the right. Anti-Traffic Jam (TJ) marks the SGPs (green); anti-pMoe (red) and anti-Vasa (PGCs; blue). (C-C”‘) *rib*^*+/-*^ gonad (*rib*^*35*.*14/+*^ or *rib*^*55*.*25/+*^). Eight stage 14/15 gonads were examined and a representative result is shown. (C-C”‘) *rib*^*35*.*14/55*.*25*^ gonad. Thirteen stage 14/15 gonads were examined and a representative result is shown. Settings on the confocal microscope were held constant for detection of Crb and pMoe in all images. All images are to the same scale. Scale bar: 10μm.
(TIF)
