# Supplementary figures and images for: Regulation of Gonad Morphogenesis in Drosophila melanogaster by BTB Family Transcription Factors
Source: PLoS One. 2016 Nov 29;11(11):e0167283. doi: 10.1371/journal.pone.0167283 (PMC5127561; doi:10.1371/journal.pone.0167283)

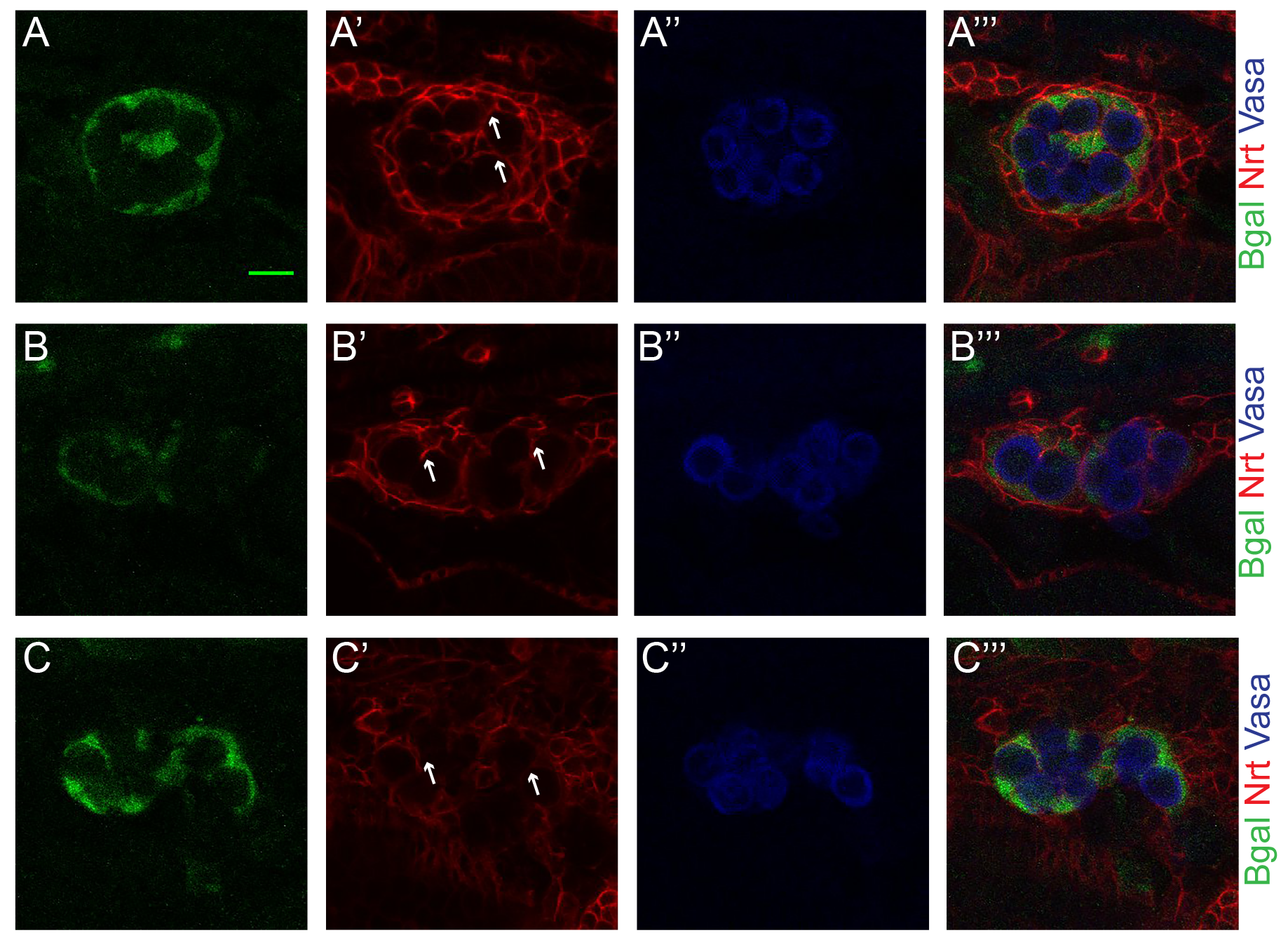

Supplement: S1 Fig — (A-A”‘) Control stage 15 embryo expressing the 68-77-lacZ enhancer trap. SGPs are labeled by anti-β-galactosidase (βgal; green); anti-Neurotactin (Nrt) labels the cell surface of somatic cells (red); and anti-Vasa labels the primordial germ cells (PGCs; blue). Arrows indicate SGP extensions ensheathing the PGCs. Scale bar: 10μm. (B-B”‘) lola46.38/22.05 stage 15 embryonic gonad exhibiting compaction defect. Cells are labeled with anti-βgal (SGPs; green), anti-Nrt (red), and anti-Vasa (PGCs; blue). Arrows indicate SGP extensions ensheathing the PGCs. (C-C”‘) rib35.14/55.25 stage 15 embryonic gonad exhibiting fusion defect. Cells are labeled with anti-βgal (SGPs; green), anti-Nrt (red), and anti-Vasa (PGCs; blue). Arrows indicate SGP extensions ensheathing the PGCs. All images are to the same scale and are a Z-projection from a stack of confocal images. (TIF) [file pone.0167283.s001.tif]

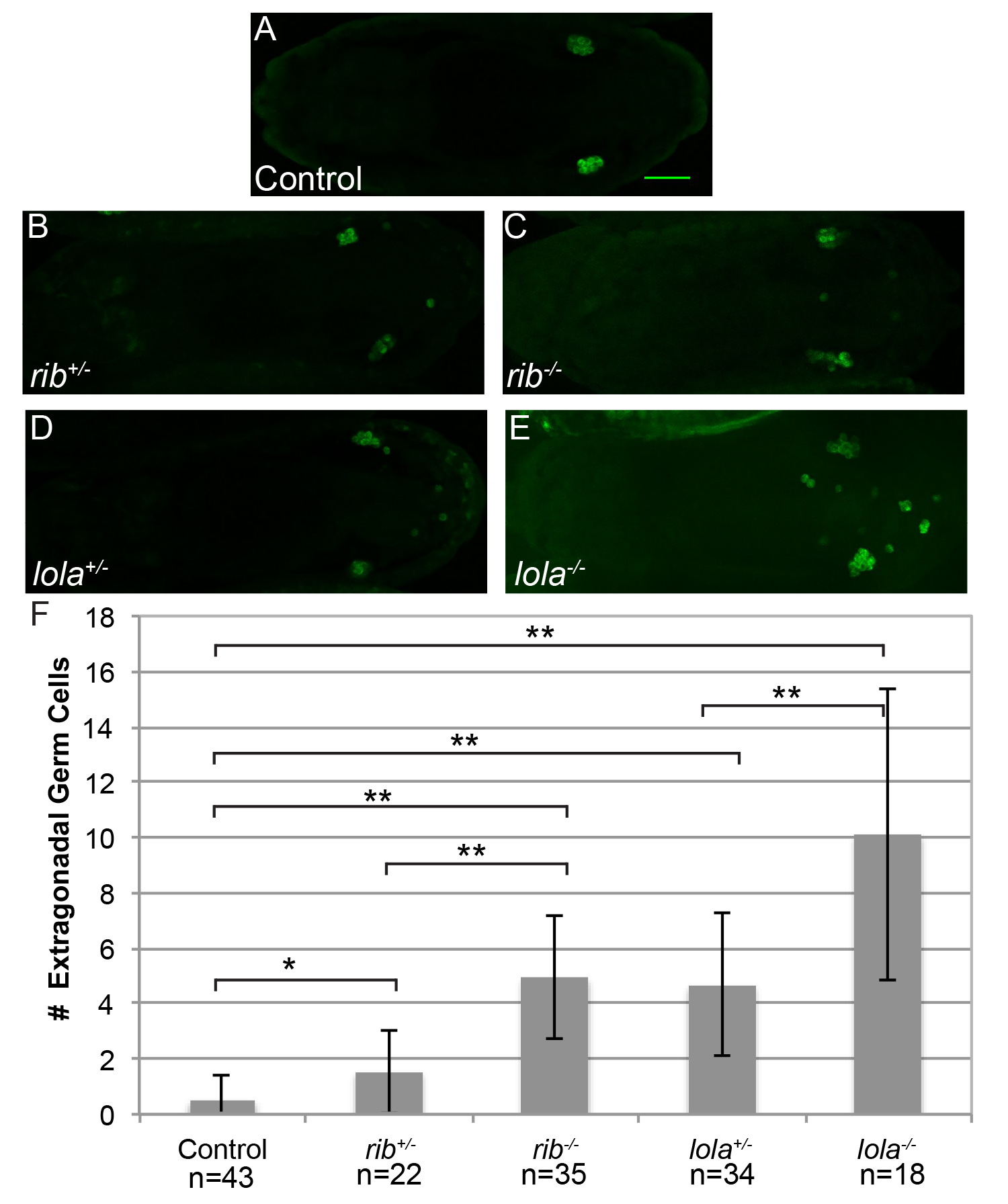

Supplement: S2 Fig — Stage 14 and 15 embryos were scored for the number of germ cells that fail to coalesce with SGPs during gonad morphogenesis. Embryos were immunostained with anti-Vasa to label the primordial germ cells and anti-GFP for genotyping. Representative embryos from the following genotypes are shown: (A) Control (68-77-lacZ), (B) rib+/- (rib55.25/+ or rib35.14/+), (C) rib-/- (rib55.25/35.14), (D) lola+/- (lola46.38/+ or lola22.05/+), and (E) lola-/- (lola46.38/22.05). (F) Quantification of the average number of extragonadal germ cells in the genotypes described above. A two-tailed, unpaired student t-test was performed to test the significance and results are noted as follows: * = p<0.01; ** = p0.001. (TIF) [file pone.0167283.s002.tif]

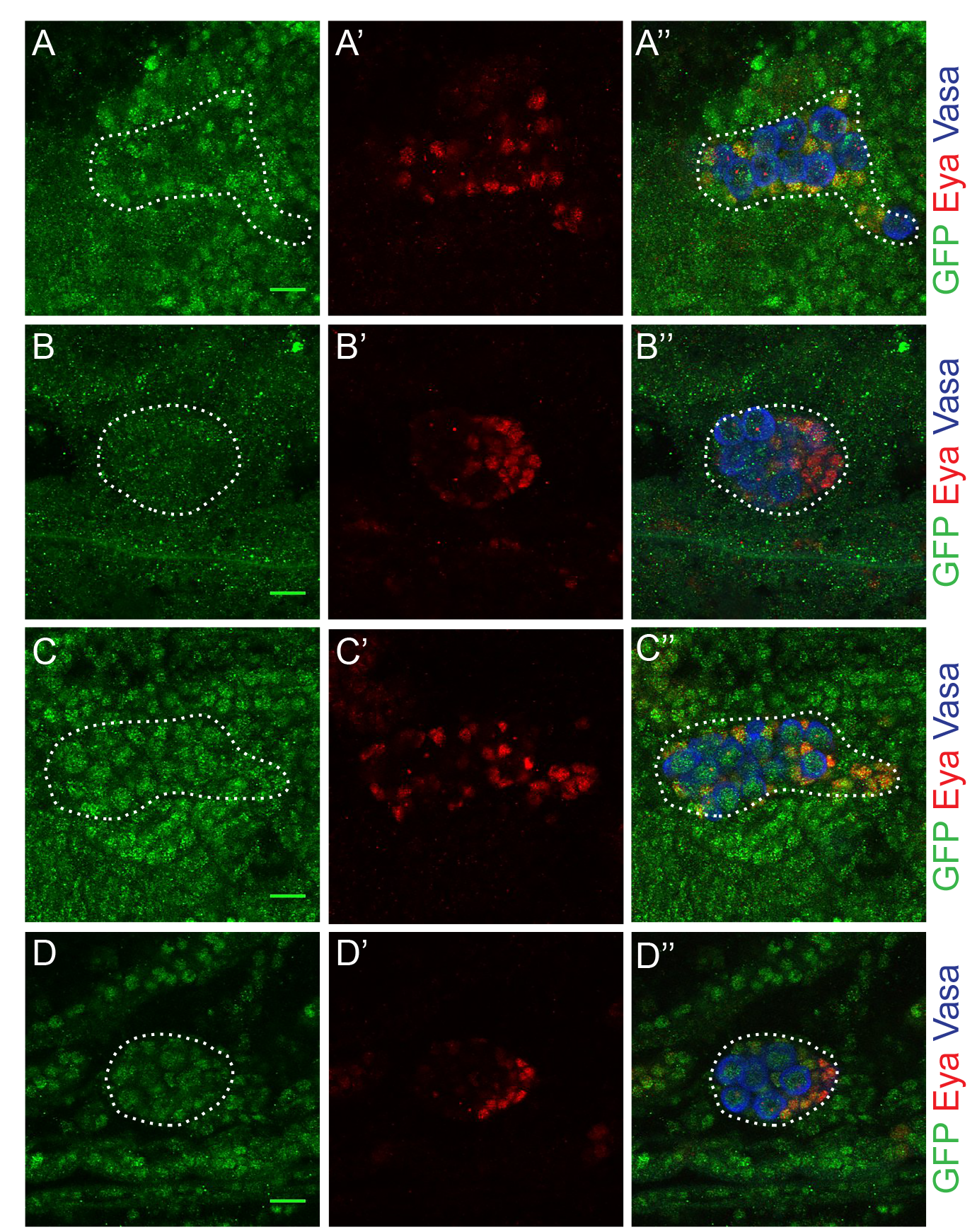

Supplement: S3 Fig — (A-A”) Expression of Lola-T in a stage 13 gonad. Image is representative of 6/6 gonads. (A) Lola-T (anti-GFP; green). (A’) Anti-Eyes absent (Eya) marks somatic gonadal precursors (SGPs; red). (A”) Merged image with anti-Lola (anti-GFP; green), anti-Eya (SGPs; red), and anti-Vasa (PGCs; blue). (B-B”) Expression of Lola-T in a stage 15 gonad. Image is representative of 11/11 gonads. (B) Lola-T (anti-GFP; green). (B’) Anti-Eya (SGPs; red). (B”) Merged image with anti-Lola (anti-GFP; green), anti-Eya (SGPs; red), and anti-Vasa (PGCs; blue). (C-C”) Expression of Lola-K in a stage 13 gonad. (C) Lola-K (anti-GFP; green). Image is representative of 13/13 gonads. (C’) Anti- Eya (SGPs; red). (C”) Merged image with anti-Lola (anti-GFP; green), anti-Eya (SGPs; red), and anti-Vasa (PGCs; blue). (D-D”) Expression of Lola-T in a stage 15 gonad. (D) Lola-T (anti-GFP; green). (D’) Anti-Eyes absent (EYA) marks somatic gonadal precursors (SGPs; red). Image is representative of 9/9 gonads. (D”) Merged image with anti-Lola (anti-GFP; green), anti-EYA (SGPs; red), and anti-Vasa (PGCs; blue). The gonad is outlined with a dotted line. (TIF) [file pone.0167283.s003.tif]

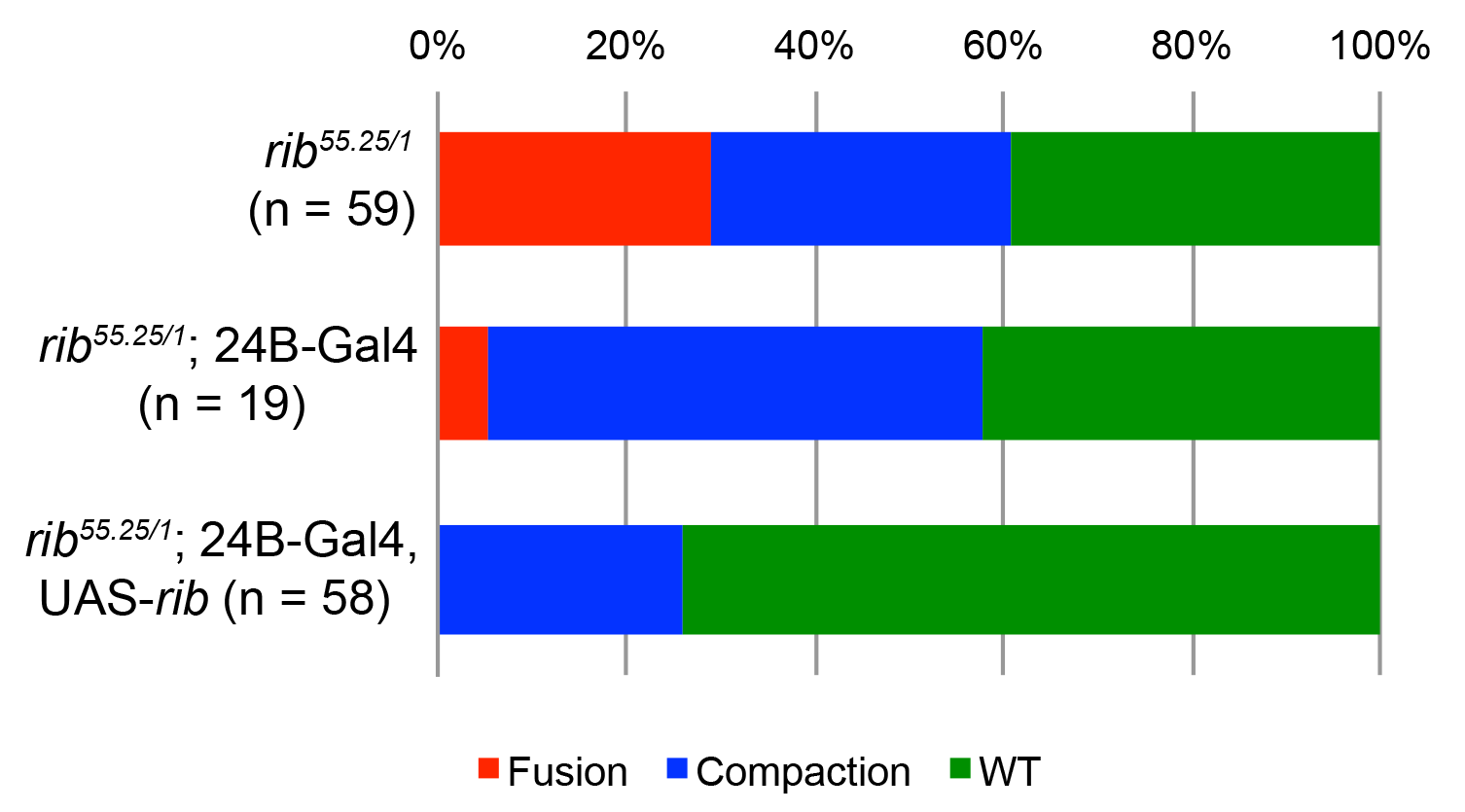

Supplement: S4 Fig — Graph of phenotypic frequency for stage 15 embryonic gonads. The following gonad phenotypes were scored: fusion (red), compaction (blue) and wild-type (green). Gonads were scored by staining somatic gonadal precursor cells for the 68-77-lacZ enhancer trap. A Chi-square test was performed to test the null hypothesis that the phenotype ratios will be the same for all genotypes. Results allow us to reject the null hypothesis: Χ2 4, 0.05 = 29.836, p<0.001. (TIF) [file pone.0167283.s004.tif]

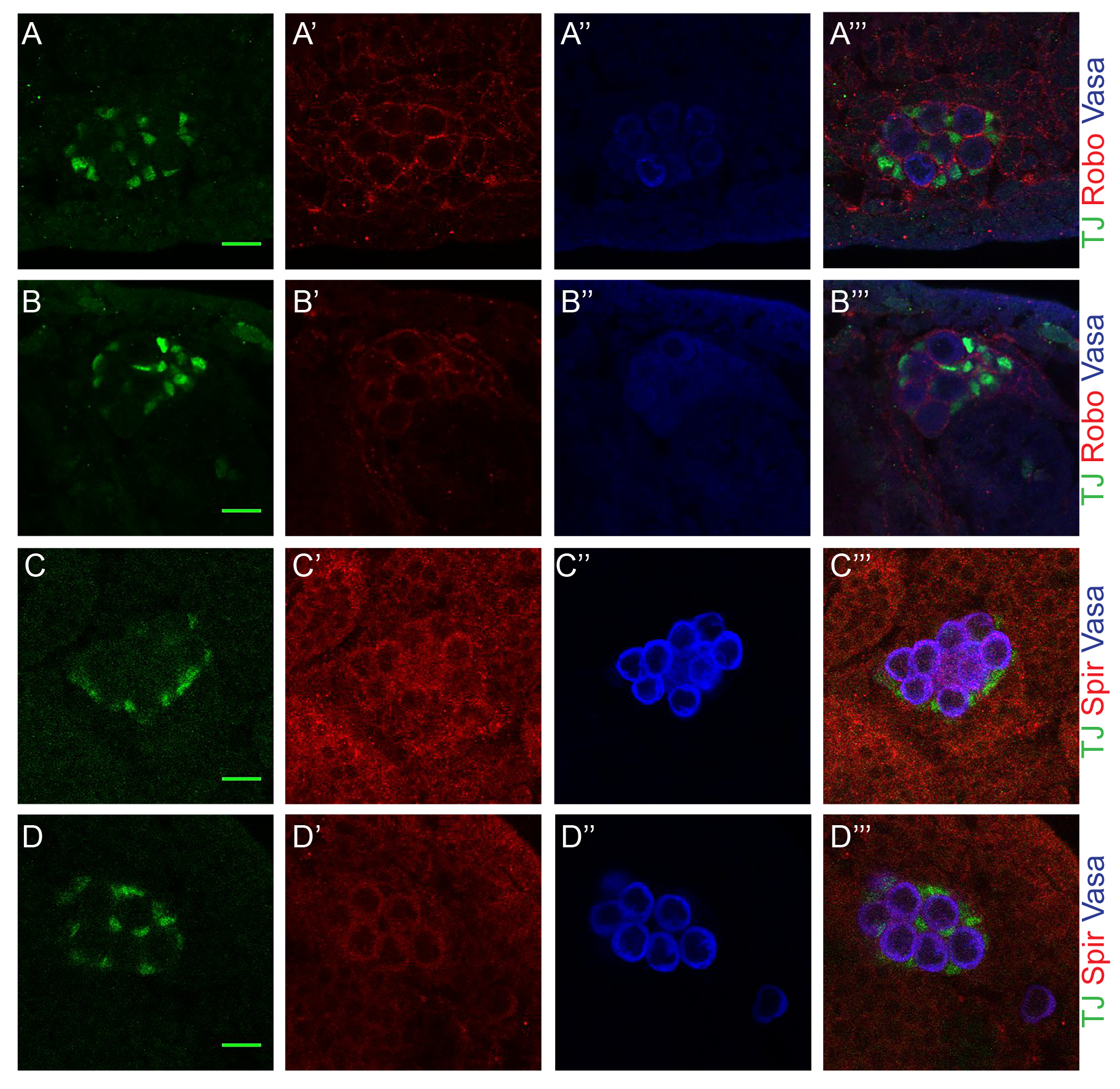

Supplement: S5 Fig — (A-B”‘) Roundabout (Robo) expression in lola heterozygous and homozygous mutant stage 15 gonads, posterior to the right. Anti-Traffic Jam (TJ) marks the SGPs (green); anti-Robo (red) and anti-Vasa marks the PGCs (blue). (A-A”‘) lolaORE76/+ gonad. Nine stage 15/16 gonads were examined and a representative result is shown. (B-B”‘) lolaORE76/ORE76 gonad. Ten stage 15/16 gonads were examined and a representative result is shown. (C-D”‘) Spire (Spir) expression in lola heterozygous and homozygous mutant stage 15 gonads, posterior to the right. Anti-TJ (SGPs; green); anti-Spir (red), and anti-Vasa (PGCs; blue). (C-C”‘) lolaORE76/+ gonad. Seventeen stage 14/15 gonads were examined and a representative result is shown. (D-D”‘) lolaORE76/ ORE76 gonad. Eighteen stage 14/15 gonads were examined and a representative result is shown. Settings on the confocal microscope were held constant for detection of Robo and Spir in all images. All images are to the same scale. Scale bar: 10μm. (TIF) [file pone.0167283.s005.tif]

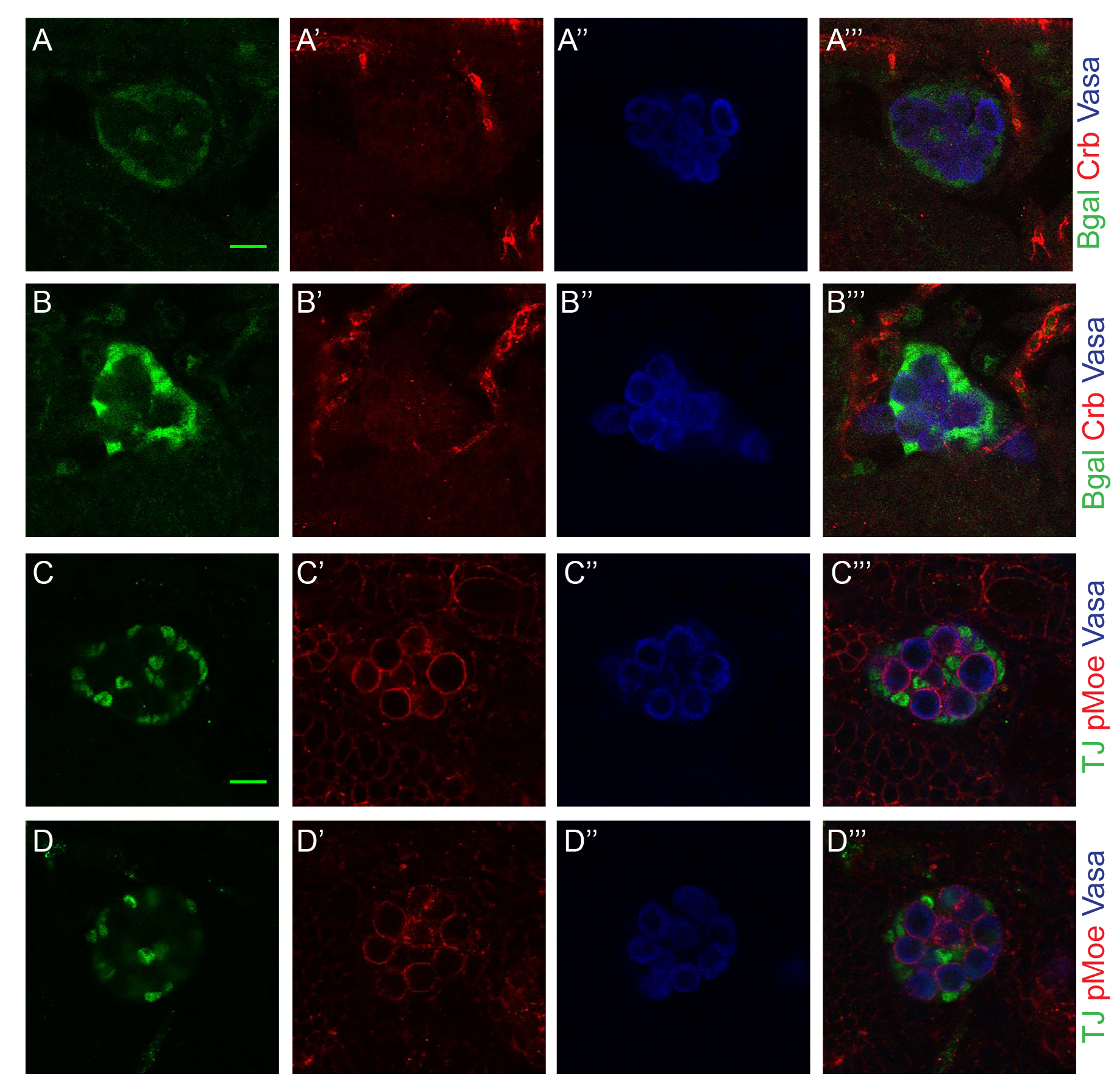

Supplement: S6 Fig — (A-B”‘) Crumbs (Crb) expression in rib heterozygous and homozygous mutant stage 15 gonads, posterior to the right. Anti-β-galactosidase (βgal) marks the SGPs due to the presence of the 68-77-lacZ enhancer trap (green); anti-Crb (red) and anti-Vasa marks the PGCs (blue). (A-A”‘) rib+/- gonad (rib35.14/+ or rib55.25/+). Twenty-one stage 14/15 gonads were examined and a representative result is shown. (B-B”) rib35.14/55.25 gonad. Thirty-one stage 14/15 gonads were examined and a representative result is shown. (C-D”‘) Phospho-Moesin (pMoe) levels in rib heterozygous and homozygous mutant stage 15 gonads, posterior to the right. Anti-Traffic Jam (TJ) marks the SGPs (green); anti-pMoe (red) and anti-Vasa (PGCs; blue). (C-C”‘) rib+/- gonad (rib35.14/+ or rib55.25/+). Eight stage 14/15 gonads were examined and a representative result is shown. (C-C”‘) rib35.14/55.25 gonad. Thirteen stage 14/15 gonads were examined and a representative result is shown. Settings on the confocal microscope were held constant for detection of Crb and pMoe in all images. All images are to the same scale. Scale bar: 10μm. (TIF) [file pone.0167283.s006.tif]
